# MolFinder: an evolutionary algorithm for the global optimization of molecular properties and the extensive exploration of chemical space using SMILES

**DOI:** 10.1186/s13321-021-00501-7

**Published:** 2021-03-18

**Authors:** Yongbeom Kwon, Juyong Lee

**Affiliations:** 1 Department of Chemistry, Division of Chemistry and Biochemistry, Kangwon National University, 1 Gangwondaehak-gil, Chuncheon, 24341 Republic of Korea; 2 Arontier Inc., 15F, 241, Gangnam-daero, Seocho-gu, Seoul, 06735 Republic of Korea

**Keywords:** Molecular optimization, SMILES, Evolutionary algorithm, Chemical space

## Abstract

Here, we introduce a new molecule optimization method, MolFinder, based on an efficient global optimization algorithm, the conformational space annealing algorithm, and the SMILES representation. MolFinder finds diverse molecules with desired properties efficiently without any training and a large molecular database. Compared with recently proposed reinforcement-learning-based molecule optimization algorithms, MolFinder consistently outperforms in terms of both the optimization of a given target property and the generation of a set of diverse and novel molecules. The efficiency of MolFinder demonstrates that combinatorial optimization using the SMILES representation is a promising approach for molecule optimization, which has not been well investigated despite its simplicity. We believe that our results shed light on new possibilities for advances in molecule optimization methods.

**Supplementary Information:**

The online version contains supplementary material available at 10.1186/s13321-021-00501-7.

## Introduction

An inverse molecular design approach, finding valuable molecules with desired properties for a given application, is drawing attention from chemists recently. Conventional molecular design approaches find novel molecules by perturbing known molecules using experienced chemists’ intuition. For validation, the designed molecules should be synthesized and tested through experiments. This whole procedure requires considerable time and resources to complete, which retards the development of novel valuable molecules. On the other hand, the inverse molecular design determines the desired properties or properties first and then searches/generates candidate molecules that are assumed to have desired properties [1, 2]. With the help of the recent development of artificial intelligence (AI)/machine learning (ML), the inverse molecular design is expected to accelerate the discovery of novel molecules in various fields including the pharmaceutical industry [3].

Various inverse molecular design methods using AI have been actively developed recently [4]. The most commonly used strategy for molecular design is to use the SMILES representation, which is a character-based linear notation in which the structure of the molecule is considered [5]. SMILES strings contain information about the structure and stereochemistry of a molecule and the presence of electric charges. Here, we briefly review a few examples of ML-based molecule generation models. First, various methods using SMILES have been developed based on the variational autoencoder (VAE) algorithm [6–8]. Recently, Zhavoronkov and coworkers successfully found novel DDR1 inhibitors using a VAE-based model [9]. VAE-based approaches convert input SMILES strings or molecular graphs into multi-dimensional vectors on a latent space based on their similarities and physicochemical properties. It is also shown that molecular transformations are possible by vector transformation on the numerical chemical space.

The second class of approaches used the recurrent neural network (RNN) algorithms [10, 11]. RNN-models are trained to learn the syntax of the SMILES representation from a large set of molecule database. After initial training, the models are used to generate novel SMILES strings sequentially. Generally, RNN-based methods have two inherent limitations. First, not all generated SMILES strings are valid; some generated strings violate the syntax of SMILES. Second, generated SMILES strings may overlap with those in the training set.

Efforts are being made to create the models that generate molecules with desired properties using the idea of reinforcement-learning (RL) [9, 11–14]. RL is an area of ML that aims to obtain the best of the selectable behaviors based on the current environment. As an example, the ReLeaSE algorithm [12] performed RL with a SMILES generating model using stacked-RNN cells [15] trained with known chemical databases. ReLeaSE was shown to generate molecules with desired physicochemical properties and was used to design possible strong binders of the JAK2 proteins. Another RL-based molecular design model is Molecule DQN (MolDQN) [13], which is based on the Deep Q-Networks (DQN) algorithm [16], which is one of the state-of-the-art RL algorithms. MolDQN uses predefined molecular variation operations to modify existing molecules into new molecules suitable for their purposes. Together with the VAE approach, RL–VAE models that improve the fitness of molecules produced by VAE via RL have been suggested [17, 18]. More comprehensive reviews of various ML-based molecular generation and optimization methods are given in detail in recent papers [4, 19–21].

The above ML-based models must be trained using existing molecular libraries such as ZINC [22], ChEMBL [23], and PubChem [24]. One potential limitation of ML-based approaches is that the results of these models heavily depend on training data. In other words, these models may be difficult to generate novel molecules that are highly dissimilar to the molecules seen during training. For example, in the case of the VAE model, the latent multi-dimensional space is constructed based on the similarities between input molecules, which guarantees good interpolation between known molecules. However, it is still not clear whether extrapolation on the latent space will yield valid molecules. In summary, ML-based models suffer from strong training data dependence, which may bias the quality and quantity of generated molecules.

In addition to recent ML-based approaches, various genetic algorithm (GA)-based molecular property optimization algorithms have been developed [25–34]. The main advantage of GA-based algorithms is that they do not require a large amount of molecule data relevant to a given optimization task because they search novel molecules in a combinatorial and stochastic way. Also, they do not need to train a molecule generator, which takes considerable computational time and resources. Most existing GA-based molecular optimization algorithms are based on the graph representation of a molecule. In recent studies, they showed competitive, sometimes better, performance compared to ML-based methods in generating novel molecules with desired properties [26, 27, 29, 30]. In addition, the design of any arbitrary operation may be limited because generally it is tightly coupled with the molecular manipulation functionality of underlying cheminformatics libraries, such as RDKit [35]. Alternative to graph-based approaches, Yoshikawa et al. proposed a GA method by converting a SMILES string into a 200-dimensional integer array based on a certain grammar [31]. However, interestingly, performing GA using the SMILES representation itself has not been well investigated despite its simplicity and computational efficiency [32, 34]. The approach has been considered less efficient than the graph-based approaches [29, 30, 33, 34].

Here, we propose the *MolFinder* method, which is a new molecular design algorithm using the conformational space annealing (CSA) algorithm [36], a class of an evolutionary algorithm. Previously, it has been considered that performing GA with SMILES is inefficient because the random crossover and the mutation operations of SMILES strings mostly result in invalid SMILES strings [29, 30]. For the global optimization of molecular properties, MolFinder employs the CSA algorithm, which has been successfully applied to many global optimization problems in various disciplines [36–40]. Compared to conventional GA, the CSA algorithm has sophisticated selection procedures to control the diversity of populations/solutions during sampling.

In this study, to show that MolFinder, a GA-based approach using SMILES, is an orthogonal and complementary approach to RL-based approaches for molecular property optimization, we compared the sampling efficiency of MolFinder with two widely used RL-based methods, ReLeaSE and MolDQN. The ReLeaSE method is one of the earliest attempts to apply reinforcement learning to find molecules with optimized properties and is being widely used. The MolDQN has also been widely used since its publication because the method uses the deep Q-Network (DQN), one of the state-of-the-art RL algorithms. The DQN method has shown its efficiency in various tasks, such as training a human-level model that plays Atari games [16]. Here, we show that MolFinder finds novel molecules with better properties than the RL-based methods, while keeping the diversity of sampled molecules. Additionally, it is demonstrated that MolFinder successfully explores a wider range of chemical space than the other RL-based methods tested here.

## Methods

### Global property optimization using conformational space annealing

The goal of this study is to develop an efficient algorithm that performs global optimization of molecular properties on chemical space. We call our method MolFinder. In this study, the CSA algorithm, a highly efficient global optimization algorithm, was utilized for the global search on chemical space [36, 38, 40–42]. CSA combines the strengths of GA, simulated annealing [43], and Monte-Carlo minimization [44]. It performs an extensive search during the initial stage of search and intensive optimization near many different local minima during the later stage of the search by controlling distance constraints between candidate solutions. The detailed description of the general CSA algorithm and its efficiency are discussed in detail elsewhere [37].

**Fig. 1:**
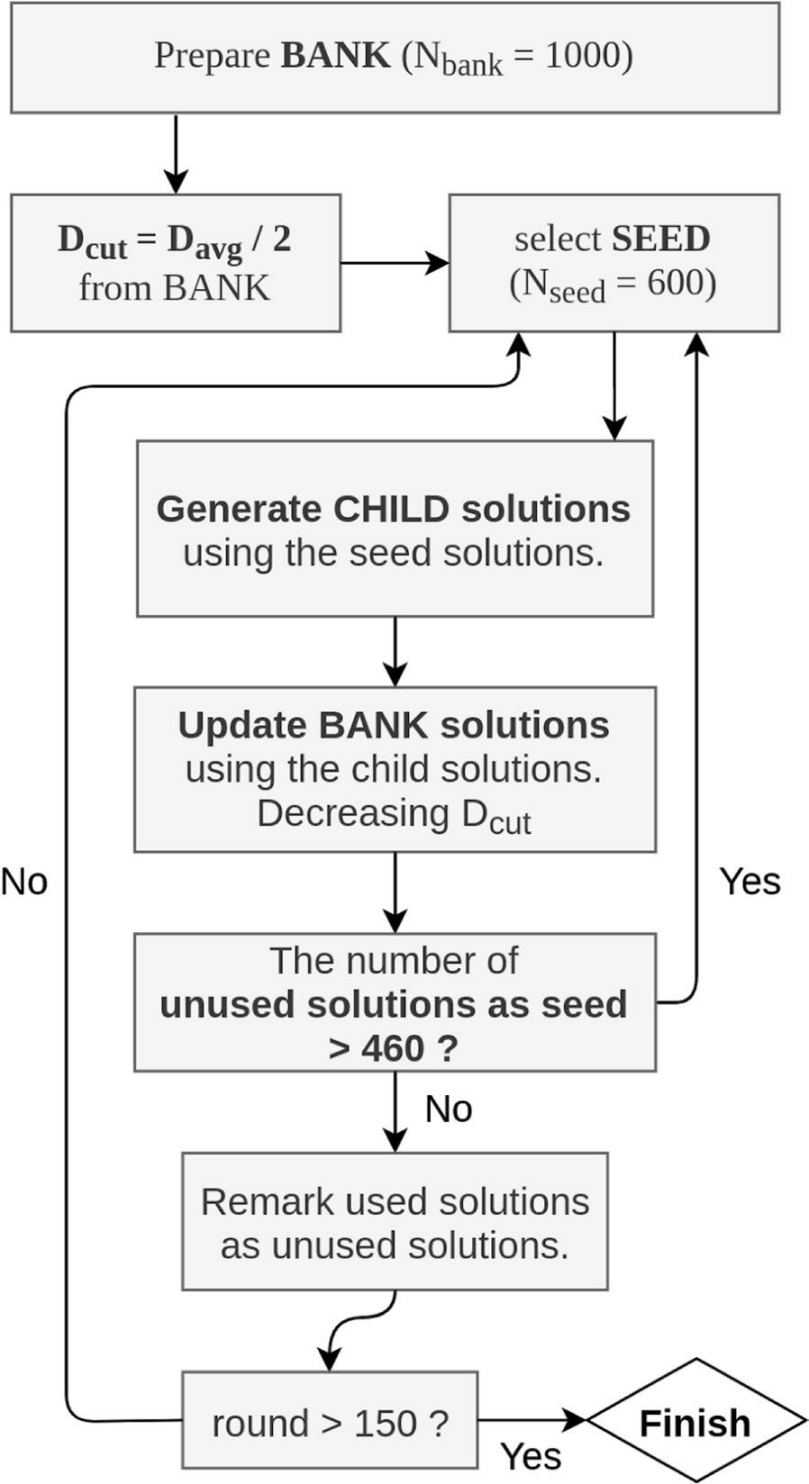
The workflow of MolFinder

MolFinder performs a global search on chemical space using the SMILES representation. The workflow of MolFinder is illustrated in Fig. 1. During the search, MolFinder uses a set of molecules called a *bank*, and its size, $N_{\text {bank}}$, is kept constant during the search. In this study, $N_{\text {bank}}$ is set to 1000. MolFinder starts with a predefined number of random molecules. The average distance between all pairs of molecules in the first bank is calculated, $D_{\text {avg}}$. The half of $D_{\text {avg}}$ is set as an initial distance cutoff, $D_{\text {cut}} = D_{\text {avg}}/2$, which is used to keep the diversity of the bank. A distance between a pair of molecules is defined as $1-S(m_{i},m_{j})$, where $S(m_i, m_j)$ is the similarity between the two molecules, $m_i$ and $m_j$. In this study, a similarity between the two molecules is calculated by using the Tanimoto coefficient of their RDKit fingerprint vectors [35].

**Fig. 2:**
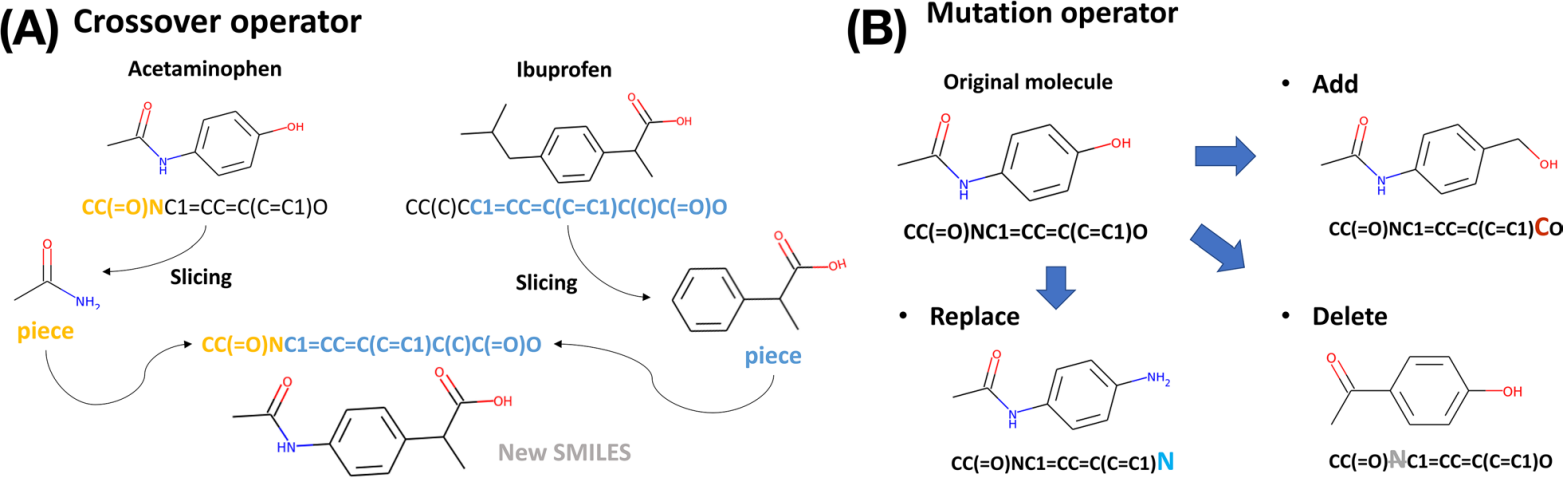
The Crossover and mutation operators The crossover (**a**) and mutation operations (**b**) using SMILES strings

Among $N_{\text {bank}}$ molecules, a subset of best molecules in terms of a given objective function with a size of $N_{\text {seed}}$ is selected as seed molecules for generating new molecules. In this study, we set $N_{\text {seed}} = 600$. Afterward, one molecule is randomly selected from this seed set, and the other from the entire bank. New molecules, child solutions, are generated from this pair through cross-over and mutation operations (Fig. 2). From a single seed molecule, 40 molecules are generated by crossover. Mutation operations consist of addition, deletion, and substitution of an atom, and 20 molecules are generated by each operation, respectively. In summary, a total of 100 molecules are generated from one seed molecule.

The generated molecules are followed by local optimization. For local optimization, atoms in a molecule are randomly substituted with other elements for a certain number of times. If the objective value of a molecule becomes better, the change is accepted, otherwise rejected. In this study, we tested the two versions of MolFinder, with and without this local optimization step. Sampling with local optimization is called MolFinder-local in this paper.

The generated new molecules are used to update the bank by considering both the diversity of molecules and their objective values. First, if a new molecule has a worse objective value than the worst of the bank, it is discarded. If it is not discarded, the molecule is compared with all molecules in the bank and its nearest neighbor is identified. Then, if the distance between the molecule and its nearest neighbor is less than $D_{\text {cut}}$, the two molecules are considered to be in the same basin on chemical space. Thus, only one molecule with a better objective value remains. If the distance between the new molecules and its nearest neighbor in the bank is larger than $D_{\text {cut}}$, the new molecule is considered to represent a favorable novel region and it replaces the molecule with the worst objective value in the bank. The $D_{\text {cut}}$ value decreases by a power of 0.98 after every generation until it reaches $D_{\text {avg}}/5$. After $D_{\text {cut}}$ becomes $D_{\text {cut}}/5$, it remains constant. By using this update procedure, the CSA algorithm enables an extensive search on chemical space and prevents the premature convergence of the search.

### Crossover operation

The key components of MolFinder are crossover and mutation operations using SMILES strings to generate novel molecules (Fig. 2). The pseudocode of the crossover operation is presented in Algorithm 1. A pair of SMILES strings are truncated from both the left and the right to enhance the diversity of substructures. In other words, one string is truncated from the left and the other from the right. The positions to be truncated are selected almost randomly for both strings by considering ring structures. To generate more valid SMILES strings, truncation of a SMILES string in the middle of a ring structure is avoided. The two truncated strings are concatenated and the numbers of open and closing parentheses are counted. If they do not match, excess parentheses are removed or deficient parentheses are inserted at random positions. After fixing imbalanced parentheses, the validity of the resulting string is checked. If the concatenated string is not valid, the procedure is repeated until it results in a valid SMILES string. If a valid SMILES is not found after 30 iterations, the left and right SMILES strings are swapped and the same procedure is repeated 30 times more. If a valid SMILES is not found, even after the additional 30 iterations, the pair is discarded. The average rate of generating a valid SMILES via crossover is 81.7% (Additional file 1: Table S1).

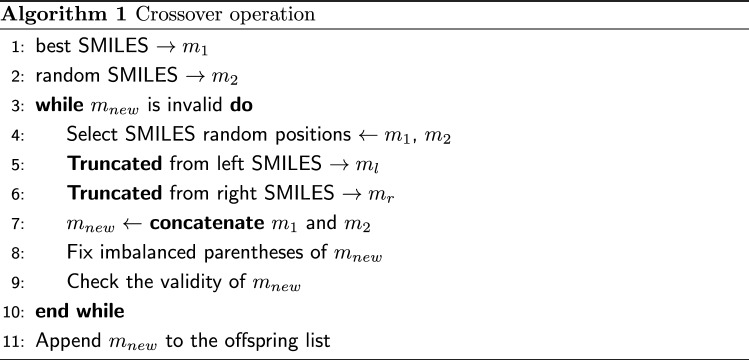



### Mutation operation

Mutation operations consist of the insertion, deletion, and substitution of atoms of a molecule. For insertion and deletion operations, an atom is inserted or deleted at a random position of a SMILES string. If the resulting string is not valid, the operation is repeated until a valid molecule is generated up to 30 times. The pseudocode of the substitution operation is shown in Algorithm 2. A random atom of a molecule is substituted with another atom considering its neighboring environment, such as the number of valences. To consider the valence of an atom properly, a SMILES string is converted to a Mol type instance of RDKit. The average rate of generating a valid SMILES via a mutation operation is over 99%.

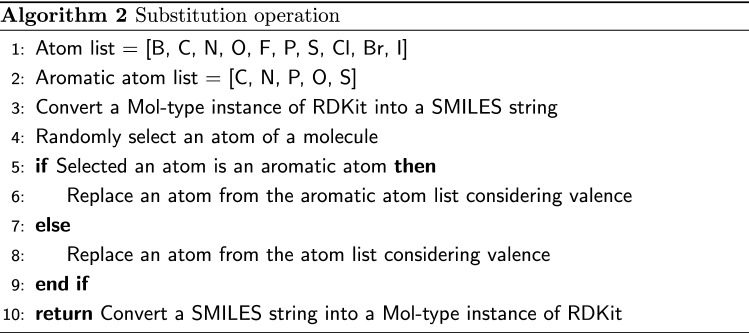



### Dataset

In this study, initial molecules were randomly sampled from the ZINC15 database [22], which consists of purchasable drug-like molecules. As of Nov. 2019, there were over 980 million SMILES strings in ZINC15 and they were grouped as tranches based on molecular weight and logP values. We randomly sampled 1/1000 of each tranche, resulting in 982,518 SMILES strings. This subset was used as a seed set for both MolFinder and the training set for other deep-learning-based generation models.

### Comparison with reinforcement-learning-based methods

To assess the efficiency of MolFinder, we compared the objective values and the diversity of generated molecules with two generative-model-based molecular property optimization approaches, ReLeaSE [12] and MolDQN [13]. ReLeaSE uses the reinforcement-learning approach [16] and a stacked-RNN model [15] to generate novel SMILES strings with desired properties. To compare with MolFinder, we used the ReLeaSE code downloaded from its Github repository [12]. The initial training of a stacked-RNN machine to learn the syntax of SMILES was performed with the training set, the random subset of ZINC15. A learning rate of 0.00005 was used. After initial training, reinforcement-learning was performed for 3000 steps to optimize the machine to produce more molecules with desired properties.

MolDQN [13] is a molecular property optimization approach based on the DQN reinforcement learning algorithm [16]. With the MolDQN approach, a seed molecule is modified by atom addition, bond addition/deletion operations to optimize target properties. The advantage of MolDQN is that it generates valid molecules mostly because it generates a new molecule by modifying a seed molecule with the predefined operations. We downloaded the MolDQN code from its Github repository and reinforcement-learning was performed for 40,000 episodes. One episode means the completion of modifying a seed molecule. Similar to ReLeaSE, MolDQN also requires the initial training of its generative model to learn the syntax of SMILES. The generator of MolDQN was trained with the identical training set with ReLeaSE. MolDQN simulations were performed from the seed molecule provided in their repository.

### Implementation detail

MolFinder was implemented with Python version 3.7.6. To compute molecular similarities and properties, RDKit version 2019.09.3.0 [35] was used. MolDQN was performed with Tensorflow version 1.15 [45] and ReLeaSE with PyTorch version 1.4 [46].

## Results and discussion

### Optimization of drug-likeness

To assess the efficiency of molecular property optimization approaches, we sampled molecules by optimizing the following objective function, a modified drug-likeness score, $S_{\text {mQED}}$:

1 $$\begin{aligned} S_{\text {mQED}}(m) = wS_{\text {QED}}(m) - (1-w)S_{\text {SA}}(m), \end{aligned}$$

where $S_{\text {QED}}(m)$ is the original quantitative estimate of drug-likeness (QED) score [47] of a molecule *m*, $S_{\text {SA}}(m)$ is the synthetic accessibility [48] of *m*, and *w* is the weight of $S_{\text {QED}}$. In this study, we used $w=0.994$. The QED score ranges from 0 to 1, and more drug-like molecules have values closer to 1. The synthetic accessibility score spans from 0 to 10, and a higher score indicates that a molecule is expected to be harder to synthesize [48]. Thus a high modified QED value, $S_{\text {mQED}}$, indicates that a molecule has similar molecular properties to known drugs and is easy to synthesize.

To assess the optimization efficiency of ReLeaSE and MolDQN, we generated 10,000 SMILES strings with each method using $S_{\text {mQED}}$ (Eq. 1). The validity of the strings was checked and only valid ones were kept for further analysis. All SMILES strings generated by MolDQN were valid. However, after removing redundancy, only 4273 molecules remained. This shows that more than half of the generated molecules by MolDQN were redundant. ReLeaSE generated 9821 valid SMILES strings from 10,000 trials. After removing redundancy, only 1340 molecules remained. In other words, more than 80% of the generated molecules by ReLeaSE were redundant suggesting that generative models may have limitations in sampling diverse molecules. For a fair comparison, the top-1000 molecules in terms of $S_{\text {mQED}}$ were selected from each generated set.

**Fig. 3:**
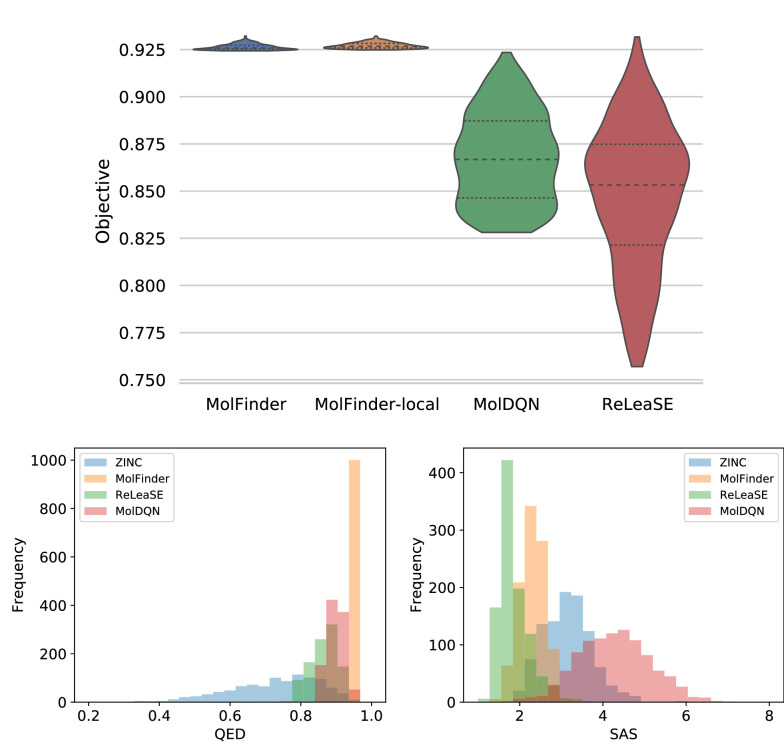
A comparison of modified drug-likeness scores of the generated molecules The violin plots of the modified drug-likeness scores of generated molecules by MolFinder, MolFinder-local, ReLeaSE, and MolDQN (top). The histogram of QED (left bottom) and SA score (right bottom) values of the generated molecules by MolFinder (orange), ReLeaSE (green), and MolDQN (red), and those of the initial ZINC15 database (blue)

A comparison of the top-1000 molecules obtained with MolFinder and the other approaches demonstrates that MolFinder discovers better molecules than the other methods (Table 1 and Fig. 3). MolFinder-local achieved the highest mean $S_{\text {mQED}}$ of 1000 molecules, 0.9240. The molecule with the highest $S_{\text {mQED}}$, 0.9326, was also obtained with MolFinder-local. It is noticeable that the minimum $S_{\text {mQED}}$ values obtained with both MolFinder models, 0.921 and 0.920, are significantly higher than those of the ReLeaSE and MolDQN results, which are 0.847 and 0.868, respectively. These numbers indicate that even the worst molecules generated by the MolFinder are comparable to those generated by the RL-based methods. When the two versions of MolFinder methods are compared, it is identified that MolFinder-local finds slightly better molecules than MolFinder.

**Table 1:** A comparison of modified drug-likeness optimization results by the MolFinder, ReLeaSE and MolDQN methods

	ZINC	MolFinder	MolFinder-local	ReLeaSE	MolDQN
Mean	0.7086	0.9237	0.9240	0.8473	0.8677
Std.	0.1248	0.0020	0.0027	0.0380	0.0240
Min.	0.3263	0.9209	0.9199	0.7570	0.8281
Max.	0.9224	0.9316	0.9326	0.9317	0.9235

Overall, the ReLeaSE results have the lowest mean and minimum $S_{\text {mQED}}$ values. However, it found one molecule that has a higher $S_{\text {mQED}}$ value than the best of MolFinder, but lower than that of MolFinder-local. This indicates that the molecules generated by ReLeaSE have a wide distribution in terms of $S_{\text {mQED}}$. Similarly, MolDQN generated a few molecules with $S_{\text {mQED}}$ values higher than 0.9. However, the $S_{\text {mQED}}$ values of most molecules generated by MolDQN were distributed between 0.85 to 0.90, which were significantly lower than the MolFinder and MolFinder-local results (Fig. 3). More details are displayed in Additional file 1: Figure S1. To show the statistical significance of this difference, we performed the two-sample t-tests by using the MolFinder results as a reference (Additional file 1: Table S2). The t-test results show that the MolFinder results have higher objective values than the RF-based methods statistically significantly.

For further analysis, we compared the distributions of the original QED score and the SA score independently (the bottom plots of Fig. 3). The analysis shows that MolFinder results have significantly higher original QED values than the other methods (left bottom of Fig. 3). All molecules generated by MolFinder had $S_{\text {QED}}$ values of higher than 0.92. On the other hand, the results of the other methods have lower $S_{\text {QED}}$ values. Following MolFinder, the most frequently observed $S_{\text {QED}}$ values of MolDQN and ReLeaSE results are centered around *0.90*. On average, MolDQN results have slightly higher $S_{\text {QED}}$ values than the ReLeaSE results. All optimization results have higher $S_{\text {QED}}$ values than ZINC15 on average.

In terms of synthetic accessibility, the ReLeaSE results have the lowest average $S_{\text {SA}}$ value meaning that they are relatively easier to synthesize, followed by the MolFinder and MolDQN results (right bottom of Fig. 3). It is noticeable that the MolDQN results have significantly higher $S_{\text {SA}}$ values than the initial molecules from ZINC15. This suggests that MolDQN tends to optimize seed molecules by modifying them into complicated and harder ones to synthesize (Additional file 1: Figure S2). On the other hand, the reinforced ReLeaSE is inclined to generate rather simpler molecules (Additional file 1: Figure S3). In summary, although both ReLeaSE and MolDQN are based on the reinforcement learning algorithms, they optimize molecules in the opposite way: making molecules simpler and more complex. The $S_{\text {SA}}$ values of MolFinder results are distributed between those of the ReLeaSE and MolDQN results, which are also improved than the ZINC15 set (Additional file 1: Figure S4, S5).

**Fig. 4:**
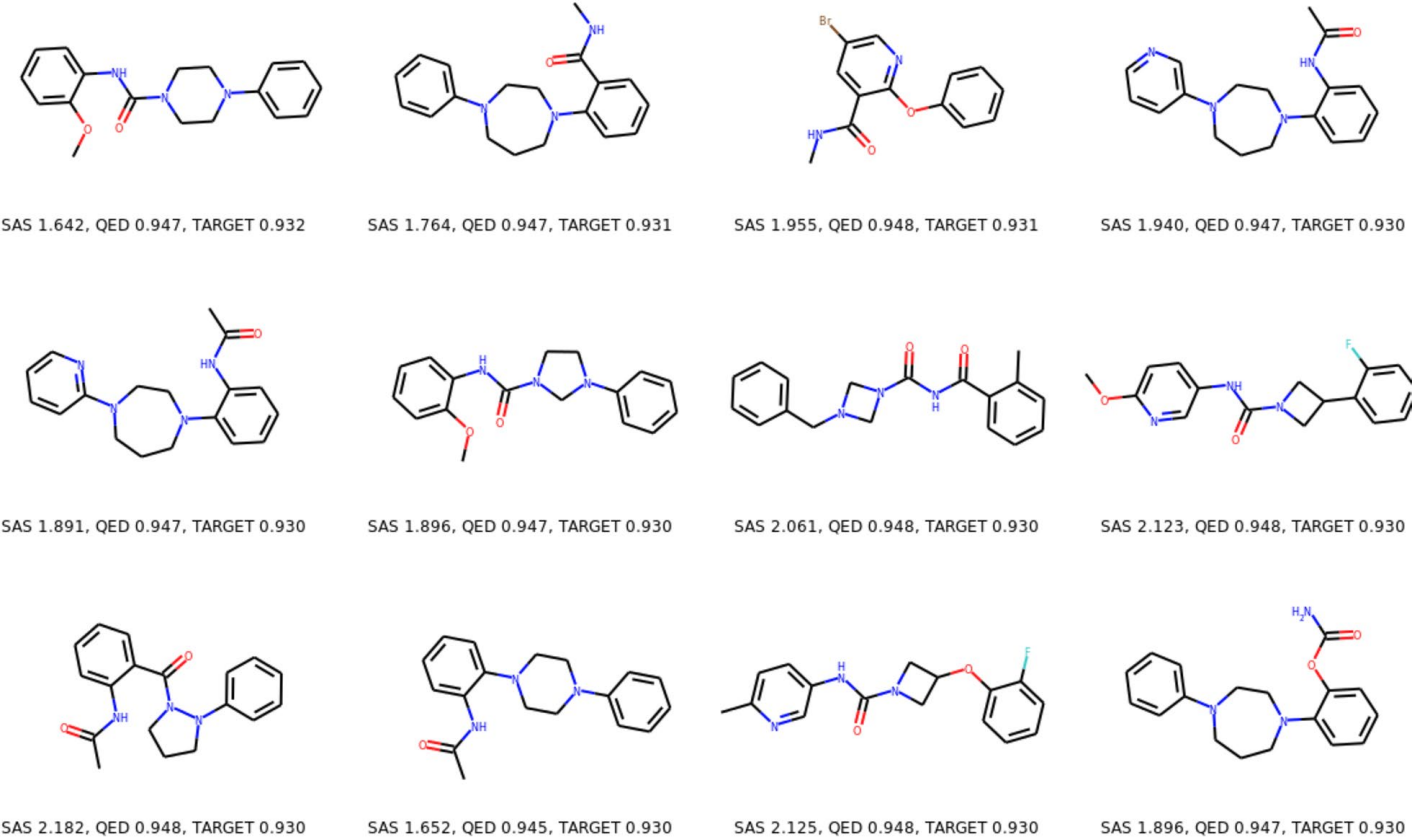
Top-12 molecules discovered by MolFinder The modified drug-likeness scores (TARGET, Eq. 1) and their drug-likeness (QED) and synthetic accessibility score (SA score) are presented

The top-12 molecules discovered by MolFinder are presented in Fig. 4. It appears that all molecules consist of relatively simple fragments and high $S_{\text {QED}}$ values. All top-12 molecules in Fig. 4 have low $S_{\text {SA}}$ values, less than 2.5, suggesting that they are readily synthesizable. It is noticeable that, even though we optimized $S_{\text {mQED}}$ in this study, the $S_{\text {QED}}$ values of the top-12 molecules are identical or comparable to the best reported values obtained from the sole optimization of $S_{\text {QED}}$ [26]. In conclusion, the above results indicate that molecule optimization of $S_{\text {mQED}}$ using MolFinder successfully generated a set of molecules with good drug-likeness and synthetic accessibility simultaneously. This clearly demonstrates that MolFinder can help accelerate the drug discovery process by generating novel drug candidates that are readily synthesizable.

Through extensive sampling of chemical space using MolFinder, we found that many different molecules have similarly high QED values. In other words, the QED measure itself has high degeneracy. This high degeneracy is due to the innate characteristics of QED design [47]. QED is calculated based on the histograms of eight representative chemical properties of 771 orally absorbable drugs. QED is designed to be scored highest if the properties of molecules correspond to the modes of the histograms. For example, the mode of molecular weight is in the range between 290 and 300 and the mode of the number of aromatic rings is two. Thus, considering the vast size of chemical space, the existence of many molecules with similar properties to known orally absorbable drugs is possible and MolFinder successfully discovered them. However, the RL-based methods have large variations in objective values because they are not fully converged to the global maximum of the objective function during optimization. When ReLeaSE and MolDQN were iterated ten times, each optimization calculation finished at quite different points in chemical space, which demonstrates that MolFinder performs more extensive sampling than the RL-based methods.

To assess whether the performance of MolFinder depends on the choice of the weight of $S_{\text {mQED}}$, we performed additional calculations with different weights (Additional file 1: Table S3). With $w=0.5$, the average $S_{\text {mQED}}$ of the top-1000 molecules obtained with MolFinder is − 0.274 while those of ReLeaSE and MolDQN are − 0.514 and − 1.101, respectively. Similarly, when $w=0.9$, the average $S_{\text {mQED}}$ of the best molecules of MolFinder results is 0.675 while those of ReLeaSE and MolDQN are 0.551 and 0.288. With $w=0.95$, the average $S_{\text {mQED}}$ of MolFinder results is 0.804 while those of ReLeaSE and MolDQN are 0.667 and 0.473. Overall, these results show that the performance of MolFinder is invariant to the change of the weights of $S_{\text {mQED}}$ and all generated molecules are unique. The structures of optimized molecules are illustrated as Additional file 1: Figures S6, S7 and S8.

We also tested optimization of the normalized form of $S_{\text {mQED}}$, $S^{\prime }_{\text {mQED}}$, with various weights. The normalized $S_{\text {mQED}}$ is defined as follows so that the contribution of SA-score is scaled from 0 to 1:

2 $$\begin{aligned} S^{\prime }_{\text {mQED}} = wS_{\text {QED}}(m) + (1-w)(1 - (S_{\text {SA}}(m) - 1)/9). \end{aligned}$$

The structures of best molecules obtained with different weights are illustrated as Additional file 1: Figures S9 to S13. When the weight is high, i.e., $w>0.9$, the molecules with high objective values are similar to those obtained with Eq. 1. When the weight is low, i.e., synthetic accessibility is considered more importantly, most molecules have rather simple chemical structures and are highly similar to each other. For example, with $w=0.1$ and 0.3, all highest-scored molecules have only two benzene rings connected with three or four bonds (Additional file 1: Figure S9). This simplicity seems to be due to the bias of SA-score [48].

### Diversity of generated molecules

**Fig. 5:**
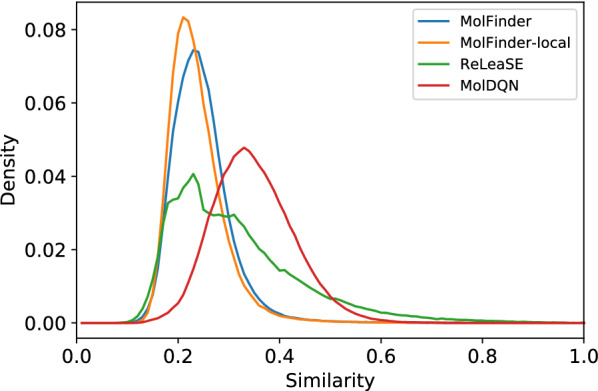
Pairwise similarities between generated molecules. The density plots of pairwise similarities between generated molecules by MolFinder (blue), MolFinder-local (yellow), ReLeaSE (green), and MolDQN (red). The pairwise similarity was calculated using the RDKit fingerprint and Tanimoto coefficient

To assess the sampling efficiency of the tested approaches, pairwise similarities between the generated molecules were investigated (Table 2). It is demonstrated that MolFinder and MolFinder-local find more diverse sets of molecules than the other RL-based approaches. This suggests that MolFinder performs a more extensive exploration of chemical space than the others. The average pairwise similarities of molecules sampled by MolFinder and MolFinder-local were 0.3106 and 0.3211, while those of ReLeaSE and MolDQN were 0.4330 and 0.4097, respectively. From the histogram of pairwise similarities, it is evident that most pairs of molecules have similarity values between 0.1 and 0.4 (Fig. 5). Although the ReLeaSE results show a peak of around 0.2, which is similar to the MolFinder results, they also include many pairs of molecules whose similarities are over 0.4. The MolDQN results have a peak of around 0.38, which is significantly larger than those of the other methods. In other words, the molecules generated by the MolFinder methods are highly diverse while those generated by ReLeaSE and MolDQN are much more similar to each other. This implies that RL-based methods are likely to be biased and their results may be confined to a certain region of chemical space possibly due to training data dependency.

**Table 2:** A comparison of pairwise similarities between generated molecules by the MolFinder, ReLeaSE and MolDQN methods

	Mean	Std.
MolFinder	0.3106	0.0716
MolFinder-local	0.3211	0.1116
ReLeaSE	0.4330	0.0782
MolDQN	0.3693	0.0719

Recently, to avoid the problem of low-diversity of RL results, Blaschke and coworkers developed a memory-assisted reinforcement learning to generate diverse optimized molecules [49]. In their algorithm, the model has the memory of previously generated molecules. If a newly generated molecule is highly similar to a saved one, a reward function is penalized. In this way, the authors showed that the RL can be improved to generate more diverse molecules while optimizing a given objective.

**Fig. 6:**
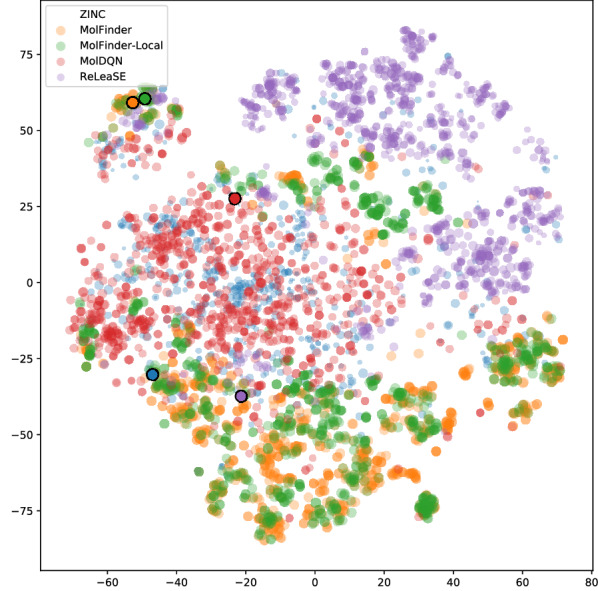
An overview of the distribution of generated molecules on chemical space The t-SNE plot of the top-1000 molecules generated by MolFinder (yellow), MolFinder-local (green), MolDQN (red), and ReLeaSE (purple). For comparison, initial/seed molecules from ZINC15 (blue) are illustrated together. The sizes of circles are proportional to the molecules’ $S_{\text {mQED}}$ values. The best molecule generated by each method is emphasized with black border lines

To identify the training/initial data dependency of the methods, the distributions of generated molecules are displayed by using the t-SNE dimension reduction method [50] (Fig. 6). A molecular similarity was calculated using the MACCS key [51]. From the plot, it is clear that MolFinder and MolFinder-local sampled different regions of chemical space compared to the initial data from ZINC15. On the t-SNE plot, MolFinder results form several distinct clusters that are widely spread over chemical space. On the other hand, molecules generated by ReLeaSE are mostly clustered at the right top of the plot, which suggests that they are similar to each other and the sampling of ReLeaSE may be biased. Also, molecules from ZINC15 are highly populated at the right top region and they are largely overlapped with the ReLeaSE results. MolDQN results overlap with the training data most. Molecules from ZINC15 and MolDQN are mostly clustered around the center and the left-center region of the plot. This indicates that molecules generated by MolDQN are highly similar to seed molecules, which may limit the sampling efficiency of the method. In summary, MolFinder and MolFinder-local explore wider regions of chemical space than the other methods.

### Assessment of novelty of molecules

To further analyze the sampling efficiencies of the molecular optimization methods, the uniqueness and novelty of molecules and their Bemis–Murcko scaffolds [52] were investigated (Table 3). Almost all molecules generated by MolFinder and MolFinder-local were novel, absent in the input database. Only one molecule generated by MolFinder was found in the input database and none by MolFinder-local. Thirty-three molecules generated by ReLeaSE were redundant. In terms of scaffolds, MolDQN found the most unique scaffolds, 880. However, as identified by higher SA scores in Fig. 3, MolDQN results have relatively complex chemical structures, such as many fused rings, which make them hard to synthesize and less practical (Additional file 1: Figure S2). The MolFinder and MolFinder-local methods generated 860 and 828 scaffolds, respectively, which are comparable to the MolDQN results. However, their $S_{\text {SA}}$ values are significantly lower than those of the MolDQN results (Fig. 3). In other words, most molecules discovered by MolFinder were drug-like and reasonably easy enough to synthesize (Additional file 1: Figures S4 and S5). It is noticeable that ReLeaSE generated only 213 unique scaffolds, which are remarkably smaller than the other methods. Many molecules generated by ReLeaSE were identified to have similar scaffolds and only peripheral groups were different (Additional file 1: Figure S3). This suggests that the reinforced generator of ReLeaSE may be biased to yield only similar molecules, which limit the efficiency of RL-based models.

**Table 3:** A comparison of uniqueness and novelty of generated molecules and their scaffolds

Method	Unique	Novel (M)	Scaffolds (N)	*P*_scaffolds_ (N/M)^a^	Novel scaffold %^b^
ZINC	1000	–	956	0.956	–
MolFinder	1000	1000	860	0.860	99.2
MolFinder-local	1000	1000	828	0.828	98.6
MolDQN	1000	997	880	0.883	96.1
ReLeaSE	967	967	213	0.220	92.0

^a^*P*_scaffolds_ represents the fraction of distinct scaffolds.

^b^The fraction of novel scaffolds was calculated by comparison with scaffolds that are contained in the training dataset

Additionally, the percentages of novel scaffolds were investigated. If a scaffold was not found in the initial dataset, it was considered novel. MolFinder results showed the highest percentage of a novel scaffold, 99.2%. The percentages of novel scaffolds of the ReLeaSE and MolDQN results, 96.1% and 92.0%, were lower than those of MolFinder and MolFinder-local. This demonstrates that the MolFinder methods not only optimize a target property more efficiently but also perform a wider exploration of chemical space than the other methods.

### Computational efficiency

To compare the computational efficiency of MolFinder and other RL-based methods, we compare the total runtime of each method. One of the advantages of GA-based methods over RL-based methods is that RL-based models require pre-training of a model to learn the syntax of SMILES generally, while GA-based algorithms do not. Overall, MolFinder required about 24 h to 36 h to obtain converged results depending on the weight on a single Intel Xeon Gold 6132 processor (Additional file 1: Table S4). The variation in runtime is due to the complexity of optimized molecules. When the weight of QED is small, relatively simple and similar molecules were generated, leading to less computational time to generate the fingerprints of molecules.

For the RL-based models, all deep-learning calculations were performed on a single RTX-2080Ti card. A MolDQN model required about 50 h to complete 40,000 episodes of reinforcement on average. To train a ReLeaSE model, it required about 100 h to train a generative model, which learns the syntax of SMILES and 33.5 h to perform reinforcement learning, optimization, on average. These results show that the computational efficiency of MolFinder is comparable to those of the RL-based methods.

### Guacamol benchmark

In the previous section, we showed that MolFinder samples better molecules in terms of $S_{\text {mQED}}$. However, it was suggested that optimization of QED is trivial and may not be an effective way to assess the efficiency of a molecular optimization method [29]. Thus, for a more rigorous assessment of our method, we performed the optimization of the goal-directed tasks of the Guacamol benchmark set [29]. The Guacamol benchmark consists of multiple non-trivial optimization tasks related to the optimization of physicochemical properties of drug-like molecules and provides a common ground to assess the efficiency of molecular property optimization methods.

For each Guacamol goal-directed task, we repeated MolFinder calculations ten times and obtained the maximum objective values (Additional file 1: Table S5). The results demonstrate that the optimization efficiency of MolFinder is comparable to existing state-of-the-art methods (Table 4). MolFinder successfully found the best ones, with an objective value of 1.0, for the rediscovery tasks. For the Median molecules 2 and Amlodipine multi-property optimization (MPO) tasks, MolFinder found the highest objective values than the reported values. Only for the zaleplon MPO task, MolFinder is showing a worse result than the other methods, which is probably due to the lack of a bond-order changing operation.

**Table 4:** Optimization results on the GuacaMol benchmark

Benchmark	SMILES LSTM	Graph GA	CReM	MSO	EvoMol	MolFinder
Celecoxib rediscovery	1.000	1.000	1.000	1.000	1.000	1.000
Troglitazone rediscovery	1.000	1.000	1.000	1.000	1.000	1.000
Thiotixene rediscovery	1.000	1.000	1.000	1.000	1.000	1.000
Aripiprazole similarity	1.000	1.000	1.000	1.000	1.000	1.000
Albuterol similarity	1.000	1.000	1.000	1.000	1.000	1.000
Mestranol similarity	1.000	1.000	1.000	1.000	1.000	1.000
C11H24	0.993	0.971	0.966	0.997	1.000	1.000
C9H10N2O2PF2Cl	0.879	0.982	0.940	1.000	1.000	1.000
Median molecules 1	0.438	0.406	0.371	0.437	0.455	0.412
Median molecules 2	0.422	0.432	0.434	0.395	0.417	0.454
Osimertinib MPO	0.907	0.953	0.995	0.966	0.978	0.945
Fexonadine MPO	0.959	0.998	1.000	1.000	1.000	0.999
Ranolazine MPO	0.855	0.920	0.969	0.931	1.000	0.947
Perindopril MPO	0.808	0.792	0.815	0.834	0.884	0.816
Amlodipine MPO	0.894	0.894	0.902	0.900	0.906	0.924
Sitagliptin MPO	0.545	0.891	0.763	0.868	0.966	0.948
Zaleplon MPO	0.669	0.754	0.770	0.764	0.810	0.695
Valsartan SMARTS	0.978	0.990	0.994	0.994	1.000	0.999
deco hop	0.996	1.000	1.000	1.000	1.000	1.000
scaffold hop	0.998	1.000	1.000	1.000	1.000	0.948

### Generating similar molecules to a reference

Designing novel molecules based on a specific scaffold or a core structure is a commonly used approach for molecular design. Thus, generating molecules with desired properties while preserving a specific scaffold has practical advantages. To benchmark this, we optimized the following objective function used by Zhou et al. [13] using MolFinder and MolDQN:

3 $$\begin{aligned} f(m) = wS_{\text {sim}}(m; m_{\text {ref}}) + (1-w)S_{\text {QED}}(m) \end{aligned}$$

where $S_{\text {sim}}(m, m_{\text {ref}})$ is the Tanimoto similarity between a molecule *m* and a reference molecule $m_{\text {ref}}$ calculated with the Morgan fingerprint and *w* is the weight coefficient of the similarity term. Here, we set $w=0.8$. For this test, we compared MolFinder with MolDQN because, based on the previous benchmarks, MolDQN performs much wider sampling of chemical scaffolds (Table 3).

Independent molecular generation calculations were repeated ten times using MolFinder and MolDQN based on the same reference molecule used to benchmark MolDQN (PubChem CID: 174590) [13]. Each MolDQN simulation was performed for 40,000 episodes and only the best 1000 non-redundant molecules in terms of the objective value (Eq. 3) were analyzed. Thus, 10,000 molecules were generated by MolFinder and MolDQN, respectively, and they are analyzed here.

**Fig. 7:**
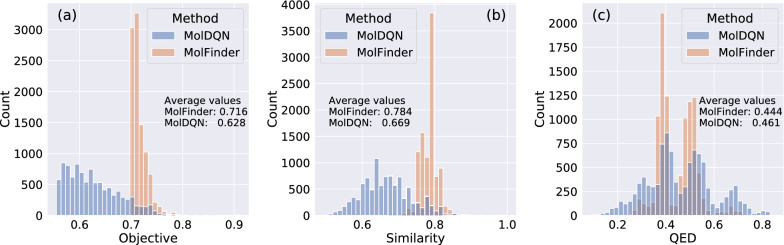
Assessment of generating similar molecules to a reference Histograms of **a** objective values (Eq. 3), **b** similarities to the reference molecules, and **c** drug-likeness scores (QED) of molecules generated by MolFinder (orange) and MolDQN (blue)

It is demonstrated that the molecules generated by MolFinder have remarkably higher objective values and similarities than those generated by MolDQN (Fig. 7). The average objective value of the MolFinder results was 0.716, while that of the MolDQN results was 0.628. All molecules generated by MolFinder have higher objective function values over 0.7, while MolDQN results peaked around 0.6.

This difference is mainly attributed to the higher similarity to the reference molecule [$S_{\text {sim}}(m; m_{\text {ref}})$ in Eq. 3]. The molecules generated by MolFinder had an average similarity of 0.784 to the reference. However, the molecules generated by MolDQN were less similar to the reference with an average similarity of 0.669. This result shows that MolFinder results are much similar to the reference as intended. In terms of the QED, the MolDQN results were slightly better than the MolFinder results, 0.461 to 0.444, while the difference is much smaller than that of the similarity. It is not clear whether such a small difference in QED, 0.017, will lead to a significant difference in the final quality of generated molecules. In summary, these results suggest that MolFinder outperforms MolDQN in generating molecules that have desired properties and are similar to a given reference molecule, simultaneously.

## Conclusion

In this study, we presented a new molecule optimization approach, MolFinder, based on the efficient global optimization of molecular properties using the SMILES representation. This method performs a global search on chemical space by using the crossover and mutation operations of the SMILES representation, which makes the method computationally efficient and straightforward to implement. Our work indicates that applying evolutionary algorithms based on the SMILES representation to molecular property optimization is promising, which has been overlooked by the field despite its simplicity. We showed that MolFinder finds better molecules than the ML-based molecular property optimization methods in terms of a given objective function. In addition, it is also demonstrated that MolFinder samples a more diverse set of molecules than the other tested methods.

The key components of the efficiency of MolFinder are the following two. First, MolFinder uses the sophisticated crossover and mutation operations of SMILES to increase the success rate of the operations. Second, the diversity of the bank of molecules was kept during the exploration of chemical space as much as possible, which is one of the key aspects of the CSA algorithm. One common limitation of conventional GA is that all solutions become highly similar to each other, meaning that the sampling is trapped in a local minimum or a set of local minima. In many previous studies using CSA, it has been shown that keeping the diversity of the bank high is critical in efficient search on multi-dimensional hyper-spaces [37–40]. However, despite the efficiency of MolFinder, we cannot completely rule out the possibility of any sampling bias caused by crossover and mutation operations.

The results presented in this paper clearly demonstrate that applying an evolutionary algorithm with the SMILES representation can be an effective strategy for molecular optimization, which is contrary to the conventional notion [27, 29, 30]. Thus our results will facilitate the development of new computational molecular design approaches based on the SMILES representation, which is advantageous in terms of its interpretability, manipulation and sharing data with other researchers. In conclusion, we believe that MolFinder is an alternative complementary approach to existing GA-based as well as ML-based methods and paves a new path for the inverse design of molecules via property optimization.

## Supplementary Information

**Additional file 1: Figure S1.** Violin plots of *S*_mQED_ optimization results using MolFinder, MolFinder-local, MolDQN and ReLeaSE. The distributions of (a) QED, (b) SA-score and (c) *S*_mQED_ values of molecular optimization calculations are displayed. ** Figure S2.** The best molecules generated by MolDQN with their modified drug-likeness values.** Figure S3.** The best molecules generated by ReLeaSE with their modified drug-likeness values.** Figure S4.** The best molecules generated by MolFinder with their modified drug-likeness values.** Figure S5.** The best molecules generated by MolFinder-local with their modified drug-likeness values. **Figure S6.** The top 15 molecules generated by MolFinder and *S*_mQED_ with weights of 0.95, 0.9 and 0.5. **Figure S7**. The top 15 molecules generated by MolDQN and *S*_mQED_ with weights of 0.95, 0.9 and 0.5. **Figure S8.** The top 15 molecules generated by ReLeaSE and *S*_mQED_ with weights of 0.95, 0.9 and 0.5. ** Figure S9.** The top 20 molecules generated by MolFinder and *S*ʹ_mQED_ with weights of 0.1 and 0.3.** Figure S10.** The top 20 molecules generated by MolFinder and *S*ʹ_mQED_ with weights of 0.5 and 0.7.** Figure S11.** The top 20 molecules generated by MolFinder and *S*ʹ_mQED_ with weights of 0.90 and 0.92.** Figure S12.** The top 20 molecules generated by MolFinder and *S*ʹ_mQED_ with weights of 0.94 and 0.96.** Figure S13.** The top 20 molecules generated by MolFinder and *S*ʹ_mQED_ with weights of 0.98 and 0.99. **Table S1.** A comparison of rates of valid SMILES generation with crossover and mutation operations. **Table S2.** Mean, standard deviation, min and max values of molecular optimization results and their two-sample t-test results. **Table S3.** Comparison of *S*_mQED_ results with different weights. **Table S4.** The list of runtime of *Sʹ*_mQED_ optimization calculations with different weights.** Table S5.** Summary and Top1 of Guacamol benchmark of MolFinder.

## Data Availability

The datasets supporting the conclusions of this article are available via https://github.com/duaibeom/MolFinder github repository.
