# Supplementary material for: MolFinder: an evolutionary algorithm for the global optimization of molecular properties and the extensive exploration of chemical space using SMILES
Source: J Cheminform. 2021 Mar 18;13:24. doi: 10.1186/s13321-021-00501-7 (PMC7977239; doi:10.1186/s13321-021-00501-7)
Supplement: Supplementary file 1 — Additional file 1: Figure S1. Violin plots of SmQED optimization results using MolFinder, MolFinder-local, MolDQN and ReLeaSE. The distributions of (a) QED, (b) SA-score and (c) SmQED values of molecular optimization calculations are displayed. Figure S2. The best molecules generated by MolDQN with their modified drug-likeness values. Figure S3. The best molecules generated by ReLeaSE with their modified drug-likeness values. Figure S4. The best molecules generated by MolFinder with their modified drug-likeness values. Figure S5. The best molecules generated by MolFinder-local with their modified drug-likeness values. Figure S6. The top 15 molecules generated by MolFinder and SmQED with weights of 0.95, 0.9 and 0.5. Figure S7. The top 15 molecules generated by MolDQN and SmQED with weights of 0.95, 0.9 and 0.5. Figure S8. The top 15 molecules generated by ReLeaSE and SmQED with weights of 0.95, 0.9 and 0.5. Figure S9. The top 20 molecules generated by MolFinder and SʹmQED with weights of 0.1 and 0.3. Figure S10. The top 20 molecules generated by MolFinder and SʹmQED with weights of 0.5 and 0.7. Figure S11. The top 20 molecules generated by MolFinder and SʹmQED with weights of 0.90 and 0.92. Figure S12. The top 20 molecules generated by MolFinder and SʹmQED with weights of 0.94 and 0.96. Figure S13. The top 20 molecules generated by MolFinder and SʹmQED with weights of 0.98 and 0.99. Table S1. A comparison of rates of valid SMILES generation with crossover and mutation operations. Table S2. Mean, standard deviation, min and max values of molecular optimization results and their two-sample t-test results. Table S3. Comparison of SmQED results with different weights. Table S4. The list of runtime of SʹmQED optimization calculations with different weights. Table S5. Summary and Top1 of Guacamol benchmark of MolFinder. [file 13321_2021_501_MOESM1_ESM.pdf]

SUPPLEMENTARY INFORMATION

MolFinder: an evolutionary algorithm for the global optimization of molecular properties and the extensive exploration of chemical space using SMILES

Yongbeom Kwon[??,??]
and Juyong Lee[??*]

[*]Correspondence:
juyong.lee@kangwon.ac.kr
[??]Department of Chemistry,
Kangwon National University, 1
Gangwondaehak-gil, 24341,
Chuncheon, Republic of Korea
Full list of author information is
available at the end of the article

Figures

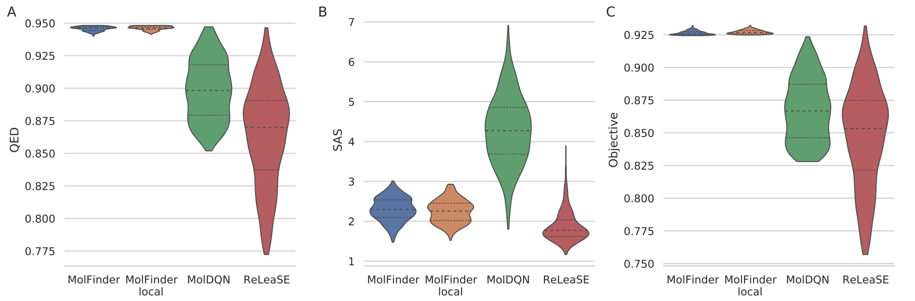

Figure S1 Violin plots of S_{mQED} optimization results using MolFinder, MolFinder-local, MolDQN and ReLeaSE. The distributions of (a) QED, (b) SA-score and (c) S_{mQED} values of molecular optimization calculations are displayed.

Table S1 A comparison of rates of valid SMILES generation with crossover and mutation operations

Method	Valid rate (%)	Time (min)
crossover (nothing)	28.340	0.441
crossover (nothing with swap)	47.434	0.720
crossover (our work)	67.451	0.974
crossover (our work with swap)	89.731	1.311
replace atom	99.99	0.909
delete atom	99.99	0.634
add atom	99.99	0.598

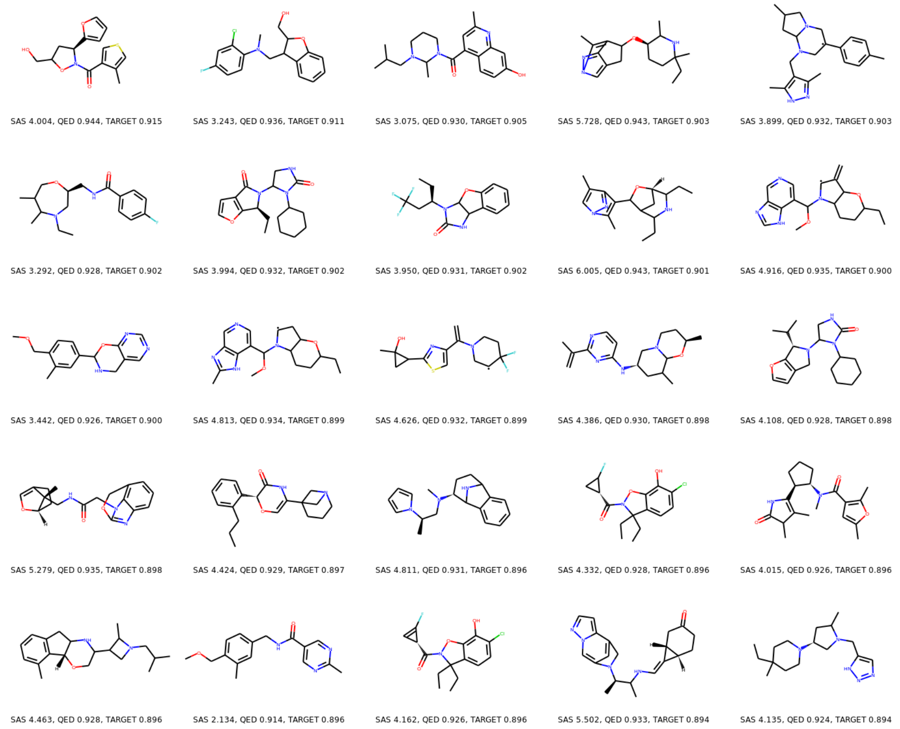

Figure S2 The best molecules generated by MolDQN with their modified drug-likeness values.

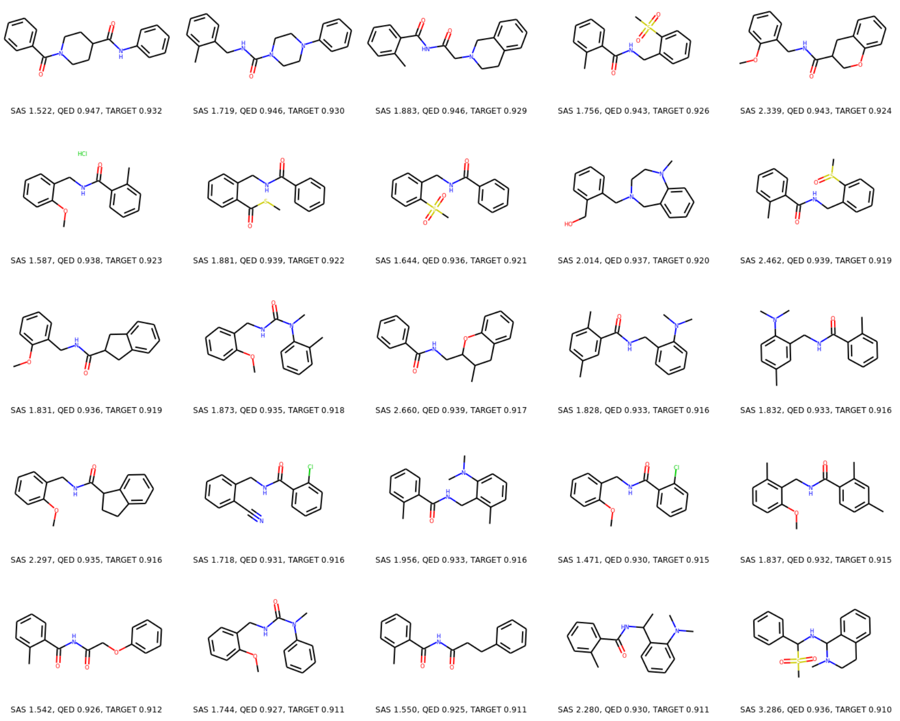

Figure S3 The best molecules generated by ReLeaSE with their modified drug-likeness values.

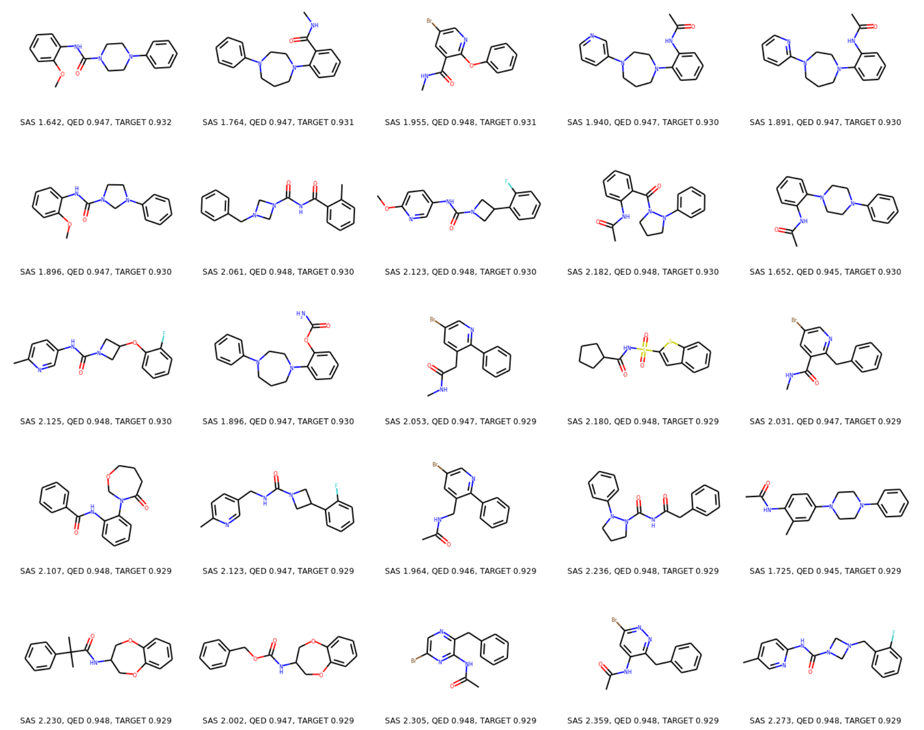

Figure S4 The best molecules generated by MolFinder with their modified drug-likeness values.

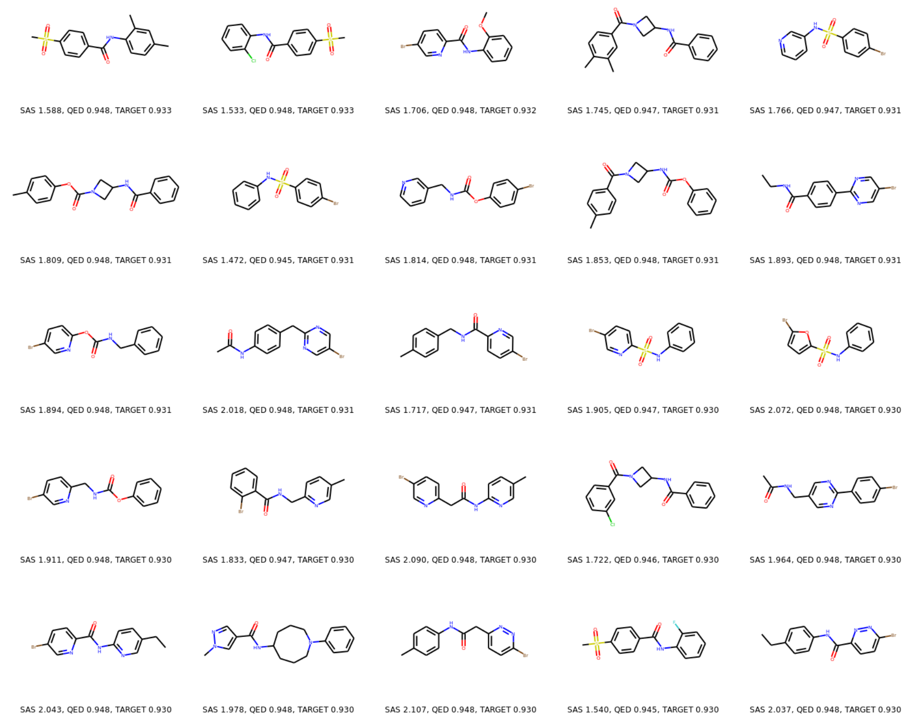

Figure S5 The best molecules generated by MolFinder-local with their modified drug-likeness values.

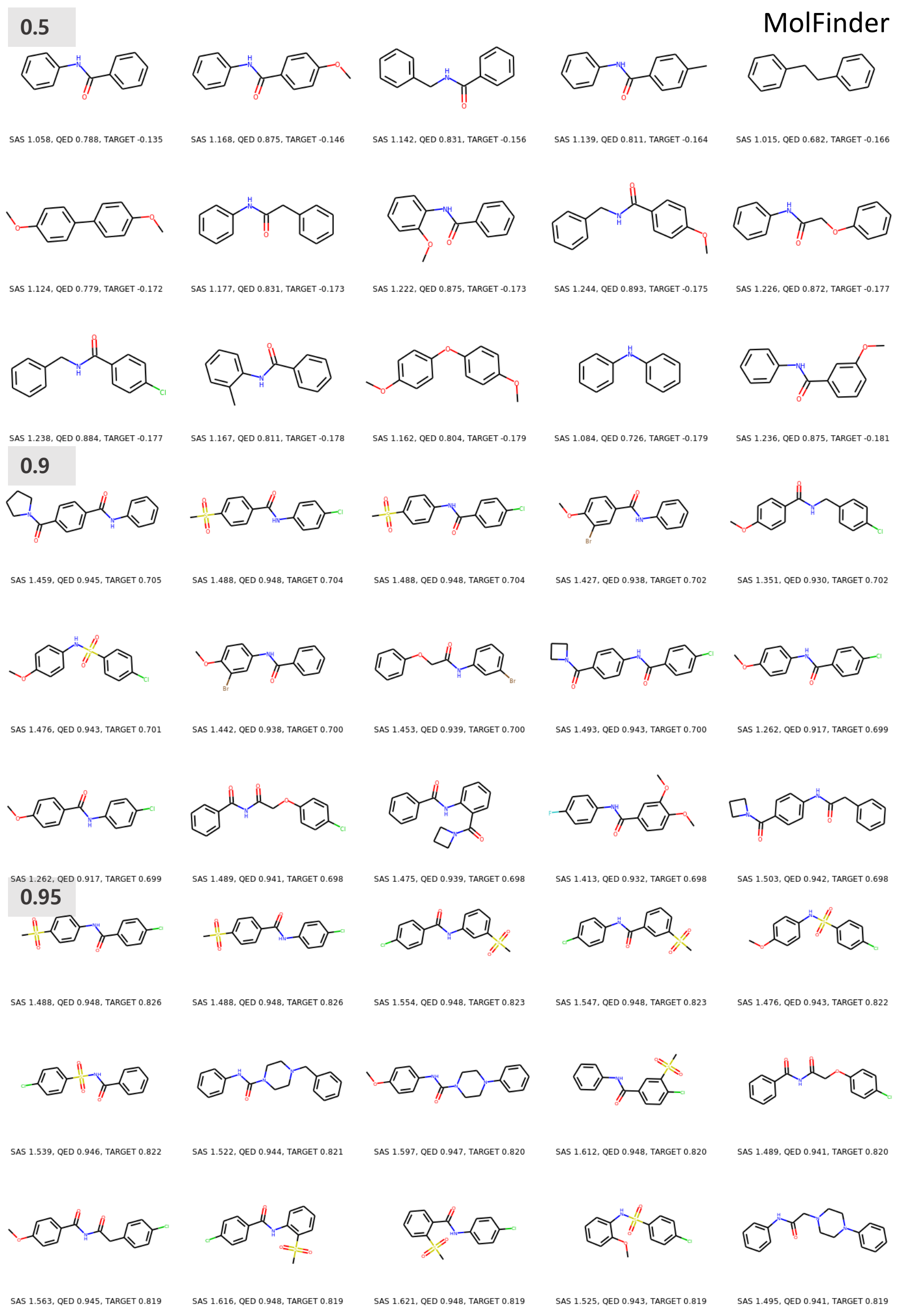

Figure S6 The top 15 molecules generated by MolFinder and S_{mQED} with weights of 0.95, 0.9 and 0.5.

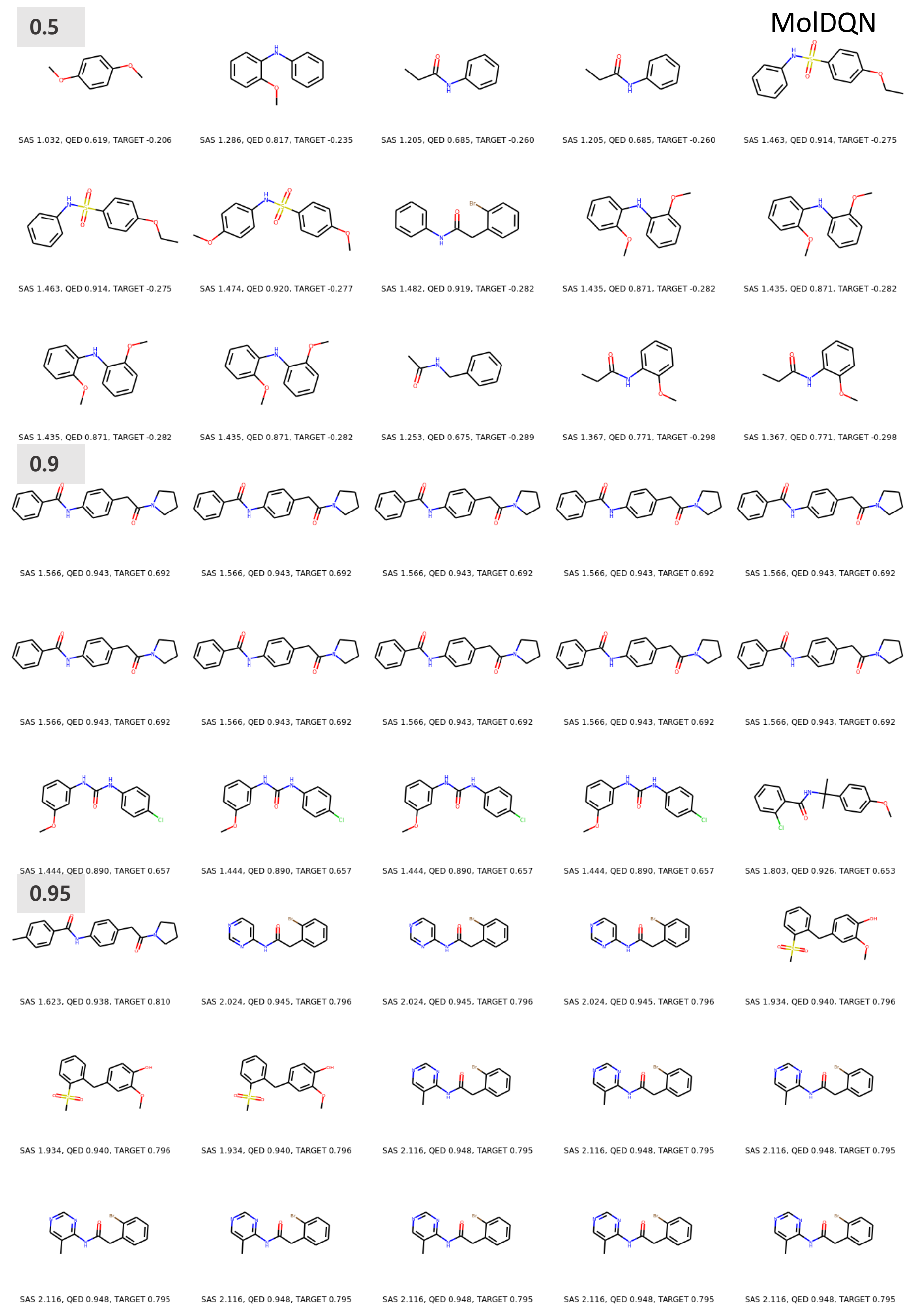

Figure S7 The top 15 molecules generated by MolDQN and S_{mQED} with weights of 0.95, 0.9 and 0.5.

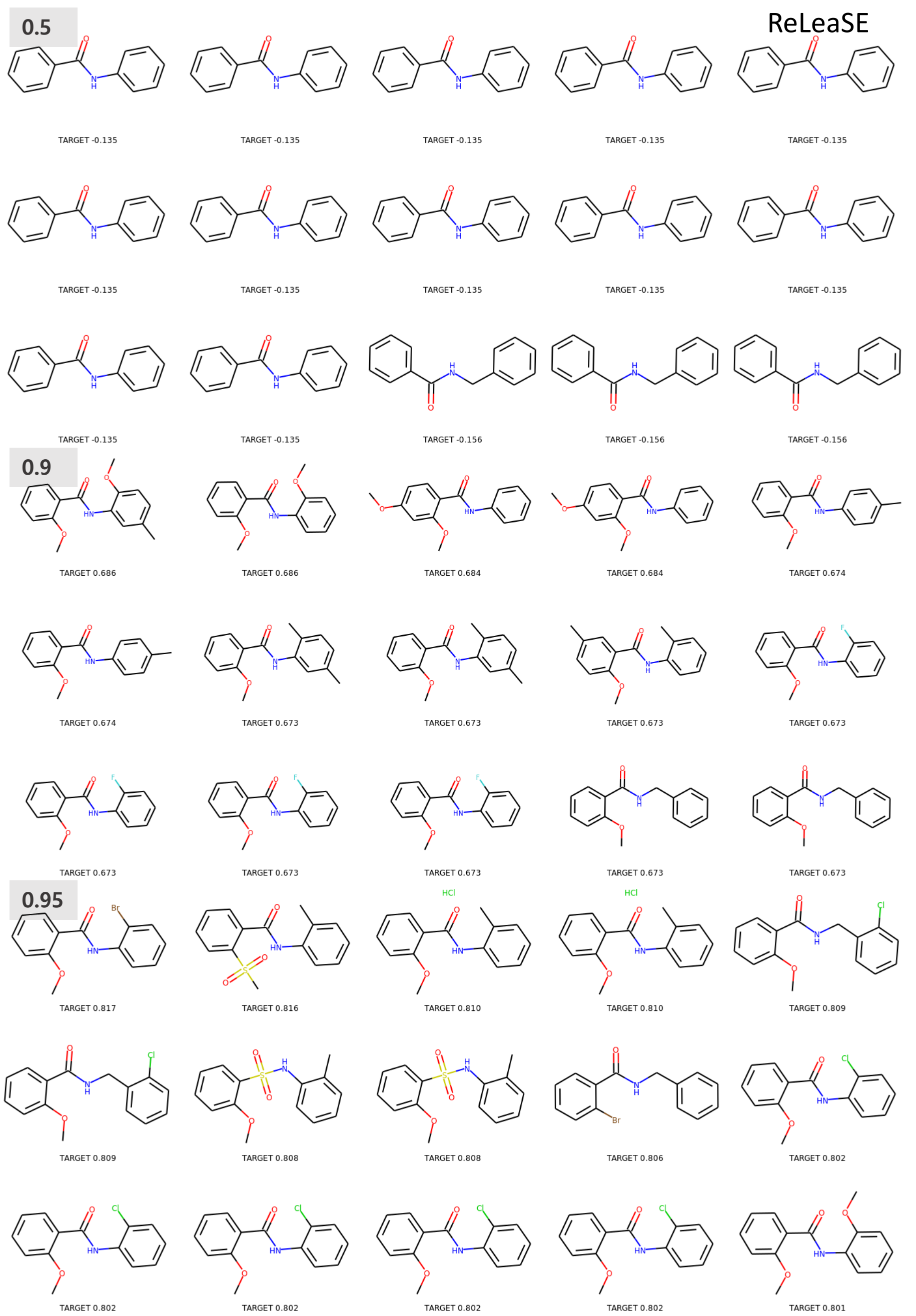

Figure S8 The top 15 molecules generated by ReLeaSE and S_{mQED} with weights of 0.95, 0.9 and 0.5.

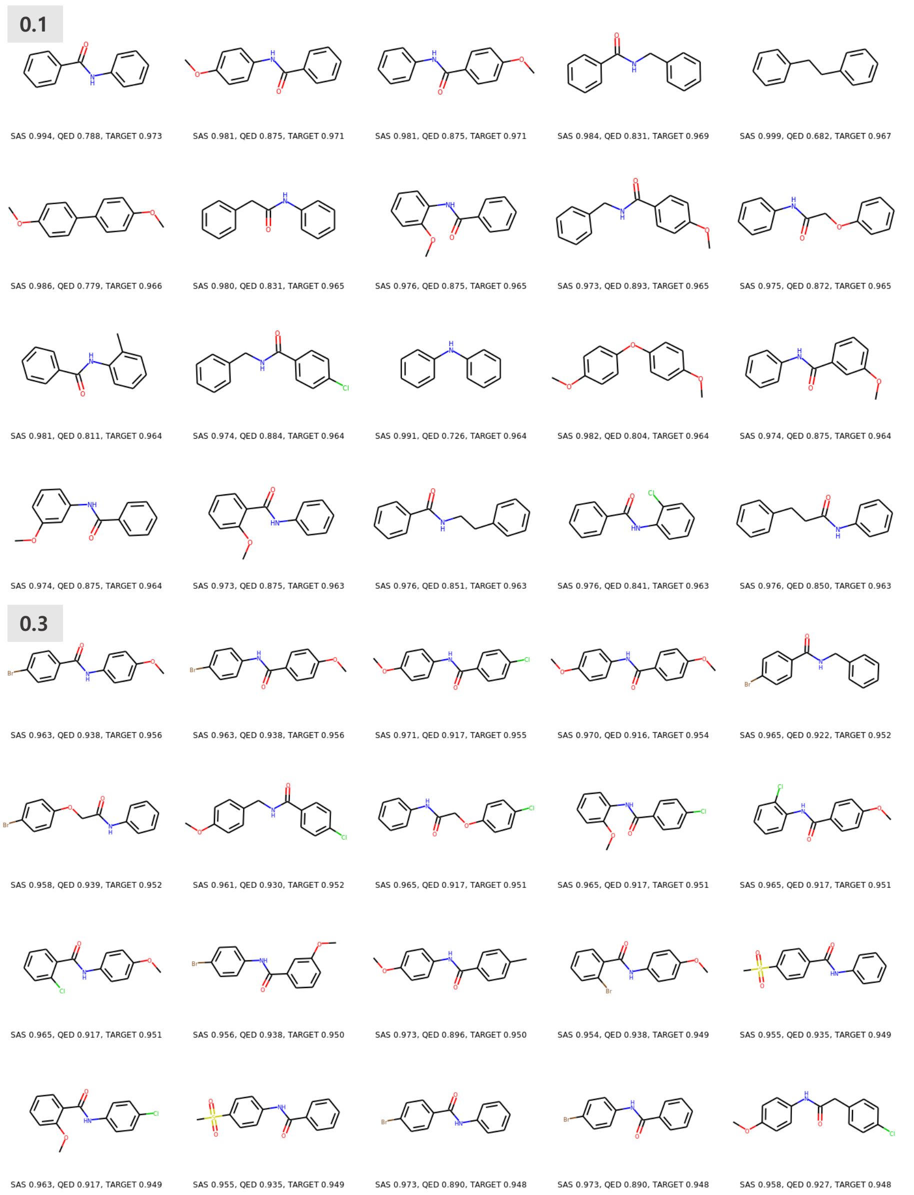

Figure S9 The top 20 molecules generated by MolFinder and S'_{mQED} with weights of 0.1 and 0.3.

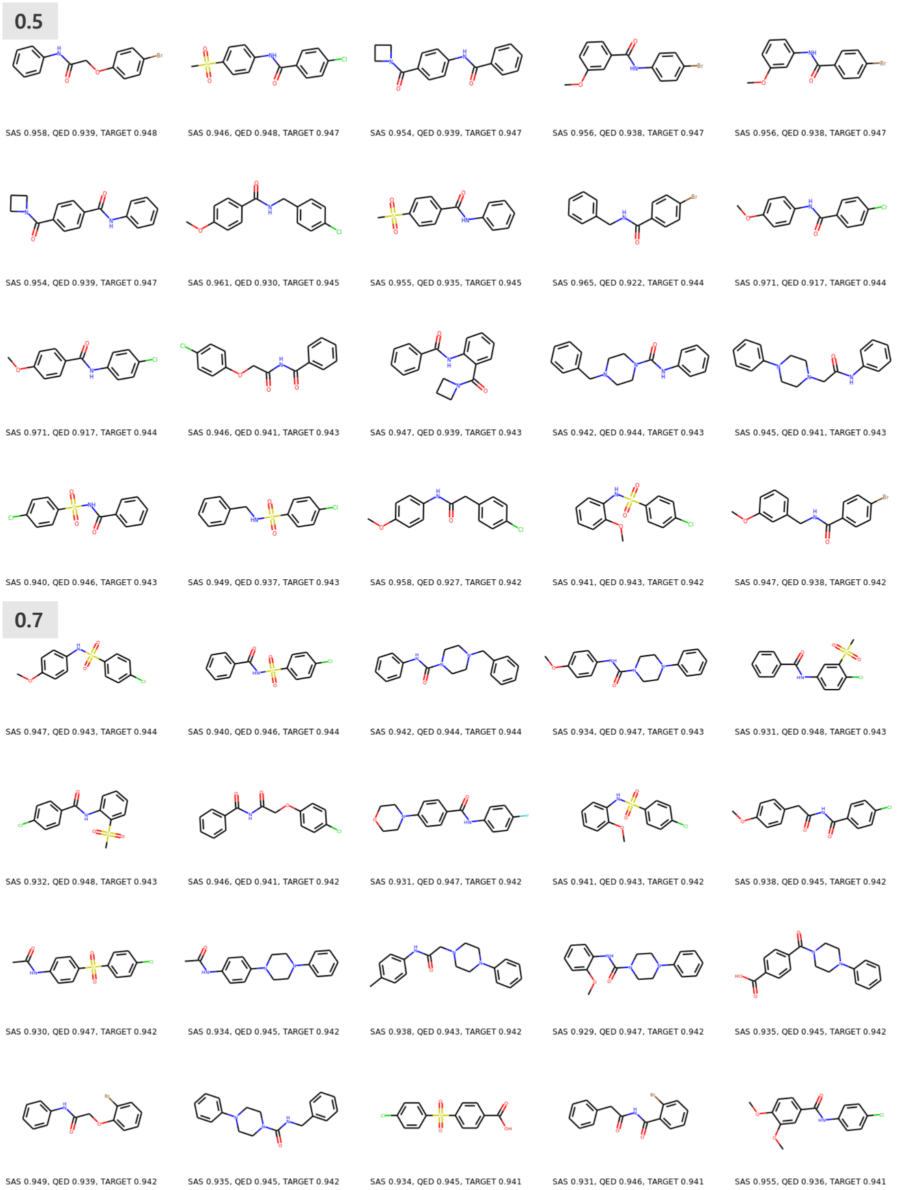

Figure S10 The top 20 molecules generated by MolFinder and S'_{mQED} with weights of 0.5 and 0.7.

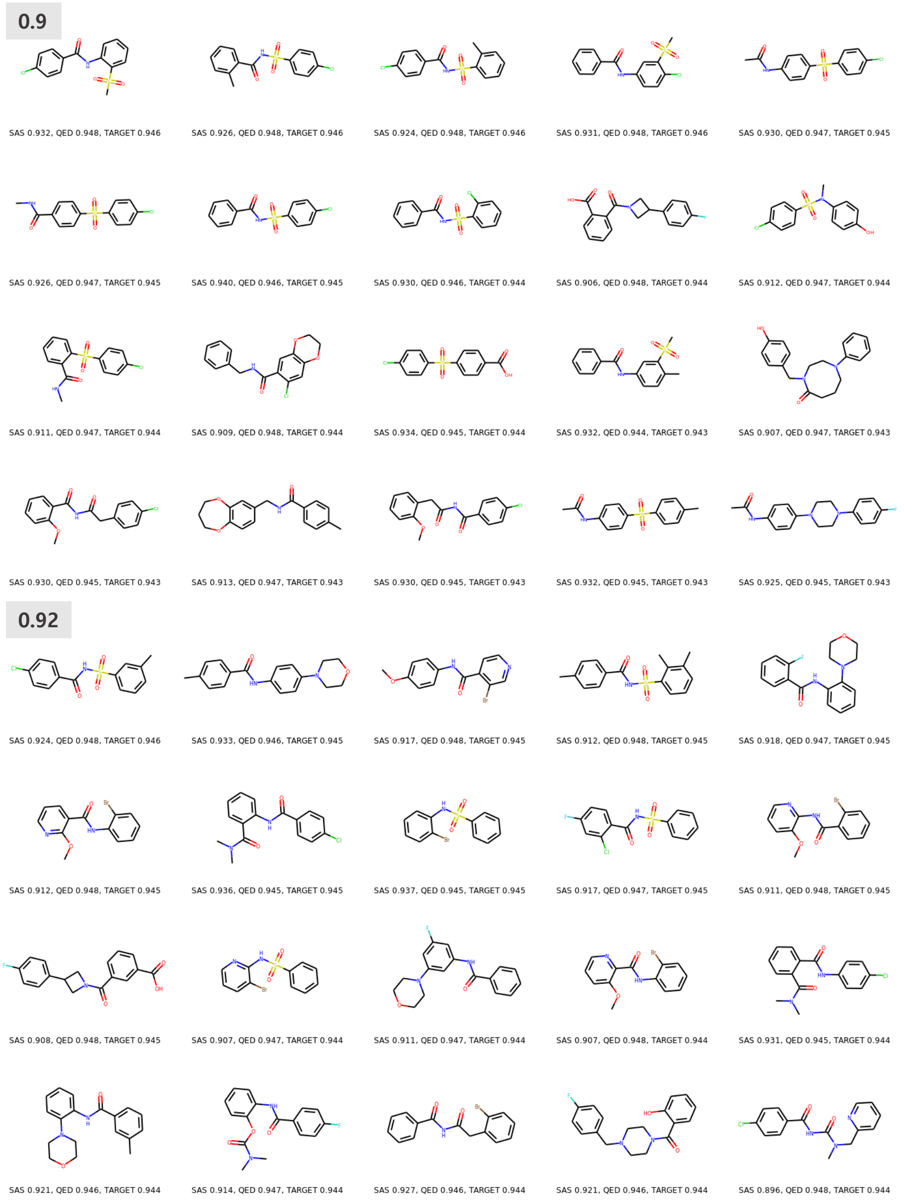

Figure S11 The top 20 molecules generated by MolFinder and S'_{mQED} with weights of 0.90 and 0.92.

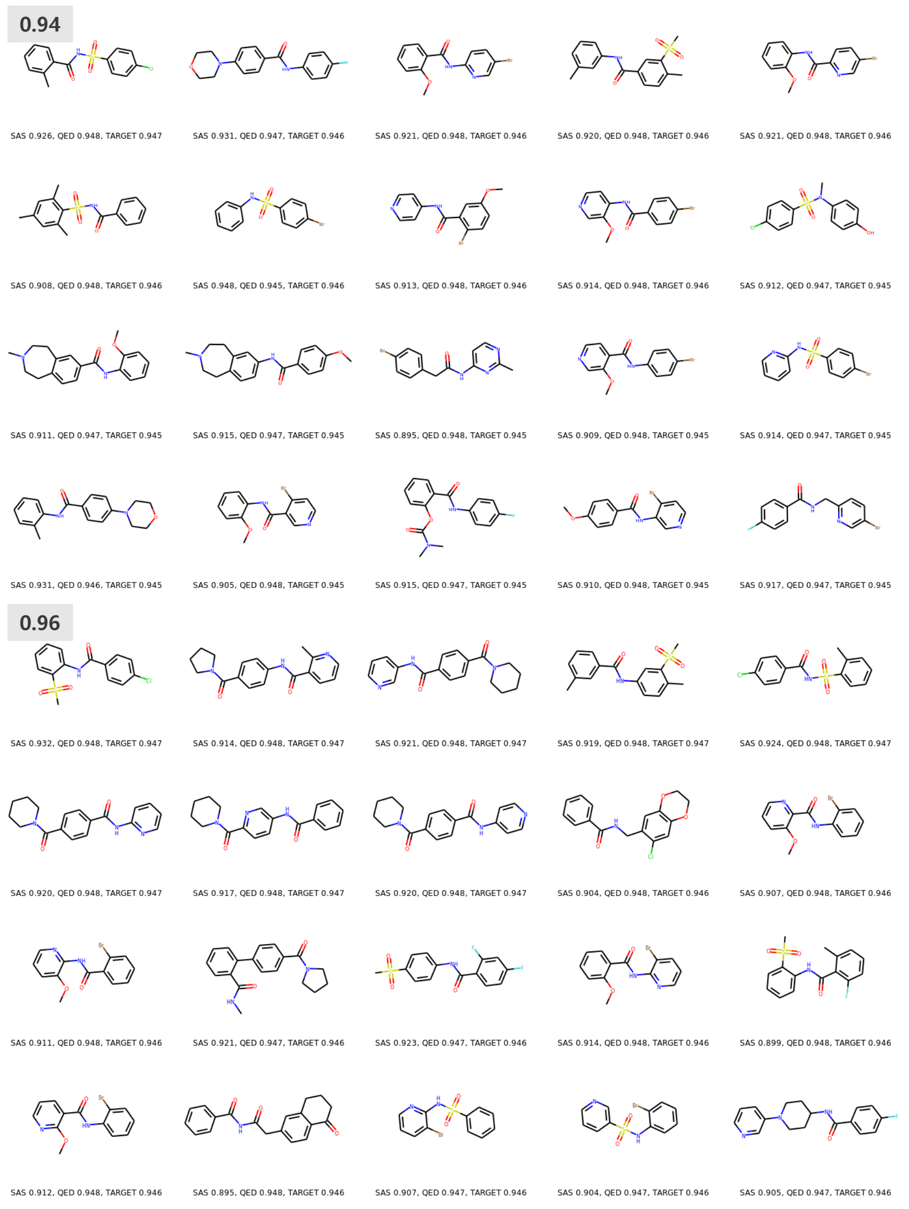

Figure S12 The top 20 molecules generated by MolFinder and S'_{mQED} with weights of 0.94 and 0.96.

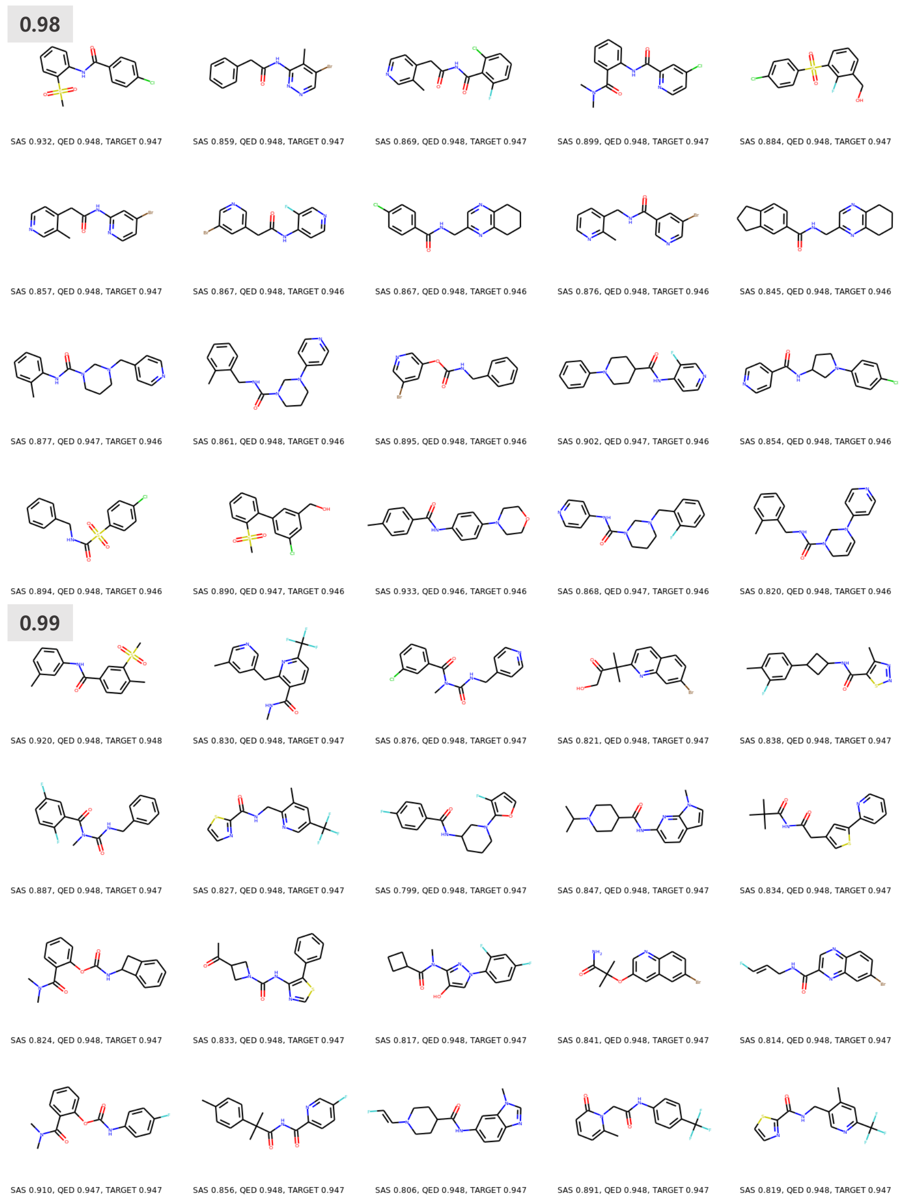

Figure S13 The top 20 molecules generated by MolFinder and S'_{mQED} with weights of 0.98 and 0.99.

Table S2 Mean, standard deviation, min and max values of molecular optimization results and their two-sample t-test results

Method		QED	SAS	Obj.
MolFinder	mean	0.946	2.30	0.926
	std	0.002	0.299	0.002
	max	0.948	3.012	0.932
	min	0.940	1.476	0.924
MolFinder-local	t-test*	3.9	-5.8	-10.6
	p-value*	1.1e-4	6.8e-9	1.6e-25
	mean	0.946	2.247	0.927
	std	0.002	0.289	0.002
	max	0.948	2.930	0.932
	min	0.941	1.522	0.925
MolDQN	t-test*	-68.2	63.9	77.1
	p-value*	0.0	0.0	0.0
	mean	0.899	4.277	0.868
	std	0.023	0.868	0.024
	max	0.947	6.912	0.923
	min	0.852	1.803	0.828
ReLeaSE	t-test*	28.1	68.3	65.7
	p-value*	0.0	0.0	0.0
	mean	0.864	1.868	0.847
	std	0.038	0.379	0.038
	max	0.947	3.892	0.932
	min	0.772	1.167	0.757

*Comparison to the MolFinder results

Table S3 Comparison of S_{mQED} results with different weights

		Obj. mean	
Method	Coefficient	top-1000 molecules	unique molecules
MolFinder	0.5	-0.223	-0.514
	0.9	0.666	0.551
	0.95	0.786	0.667
MolDQN	0.5	-0.698	-1.132
	0.9	0.581	0.288
	0.95	0.730	0.473
ReLeaSE	0.5	-0.274	-0.274
	0.9	0.675	0.675
	0.95	0.804	0.804

Table S4 The list of runtime of S'_{mQED} calculations with different weights

Coefficient	Time (day-hour:min:sec)
0.1	12:18:39
0.2	11:59:28
0.3	11:14:02
0.4	11:16:16
0.5	16:48:17
0.6	19:15:53
0.7	21:39:15
0.8	18:20:12
0.9	1-04:19:37
0.91	1-03:56:57
0.92	1-00:10:31
0.93	1-01:30:00
0.94	22:18:32
0.95	1-03:21:54
0.96	23:47:00
0.97	1-00:39:48
0.98	1-09:16:31
0.99	1-11:38:33

Table S5: Summary and Top1 of Guacamol benchmark of MolFinder

Benchmark name	Metrics	Attempts									
		1	2	3	4	5	6	7	8	9	10
Celecoxib	score	1.0	0.714	0.692	0.750	1.0	0.714	0.841	0.651	1.0	0.714
	similarity	0.472	0.484	0.472	0.450	0.471	0.478	0.634	0.509	0.458	0.485
	top1	1.0	0.714	0.692	0.750	1.0	0.714	0.841	0.651	1.0	0.714
Troglitazone	score	0.798	1.0	1.0	0.857	0.798	0.672	0.823	0.792	0.822	0.823
	similarity	0.631	0.682	0.677	0.688	0.681	0.475	0.671	0.661	0.646	0.625
	top1	0.798	1.0	1.0	0.857	0.798	0.672	0.823	0.792	0.822	0.823
Thiothixene	score	0.731	0.731	0.752	1.0	1.0	1.0	0.752	0.752	0.731	0.764
	similarity	0.437	0.499	0.520	0.648	0.520	0.670	0.496	0.511	0.504	0.587
	top1	0.731	0.731	0.752	1.0	1.0	1.0	0.752	0.752	0.731	0.764
Aripiprazole	score	1.0	1.0	0.994	0.955	0.977	0.997	1.0	0.999	1.0	0.977
	similarity	0.660	0.569	0.534	0.497	0.616	0.551	0.560	0.572	0.557	0.495
	top1	1.0	1.0	1.0	0.994	0.997	1.0	1.0	1.0	1.0	1.0
Albuterol	score	1.0	1.0	1.0	1.0	1.0	1.0	1.0	1.0	1.0	1.0
	similarity	0.288	0.289	0.290	0.301	0.275	0.284	0.293	0.286	0.301	0.265
	top1	1.0	1.0	1.0	1.0	1.0	1.0	1.0	1.0	1.0	1.0
Mestranol	score	1.0	1.0	1.0	1.0	1.0	1.0	1.0	1.0	1.0	1.0
	similarity	0.437	0.427	0.451	0.426	0.448	0.442	0.428	0.454	0.448	0.441
	top1	1.0	1.0	1.0	1.0	1.0	1.0	1.0	1.0	1.0	1.0
C11H24	score	0.964	0.964	0.964	0.964	0.964	0.963	0.964	0.963	0.963	0.964
	similarity	0.176	0.159	0.173	0.174	0.176	0.156	0.179	0.181	0.168	0.169
C9H10N2O2PF2Cl	score	0.999	0.995	0.990	1.0	0.994	1.0	1.0	1.0	1.0	1.0
	similarity	0.168	0.131	0.148	0.141	0.173	0.157	0.170	0.140	0.129	0.165
Median molecules 1	score	0.391	0.396	0.396	0.394	0.404	0.395	0.403	0.405	0.392	0.391
	similarity	0.467	0.533	0.500	0.461	0.522	0.467	0.505	0.549	0.465	0.465
	top1	0.407	0.412	0.407	0.412	0.419	0.407	0.419	0.419	0.407	0.407
Median molecules 2	score	0.413	0.412	0.392	0.415	0.415	0.414	0.408	0.415	0.415	0.408
	similarity	0.513	0.564	0.445	0.585	0.497	0.502	0.472	0.491	0.547	0.478
	top1	0.454	0.454	0.409	0.454	0.454	0.454	0.454	0.454	0.454	0.454
Osimertinib MPO	score	0.941	0.925	0.928	0.937	0.925	0.938	0.949	0.936	0.933	0.924
	similarity	0.494	0.448	0.476	0.461	0.440	0.480	0.402	0.497	0.451	0.423
	top1	0.945	0.925	0.929	0.941	0.928	0.942	0.953	0.940	0.934	0.928
Fexofenadine MPO	score	0.956	0.980	0.989	0.985	0.977	0.995	0.961	0.965	0.974	0.999
	similarity	0.564	0.455	0.510	0.465	0.456	0.508	0.414	0.400	0.488	0.514
	top1	0.966	0.987	0.999	0.999	0.995	1.0	0.978	0.979	0.982	1.0
Ranolazine MPO	score	0.928	0.943	0.940	0.923	0.920	0.930	0.936	0.927	0.925	0.911
	similarity	0.404	0.501	0.464	0.513	0.407	0.467	0.549	0.531	0.452	0.379
	top1	0.933	0.946	0.947	0.926	0.924	0.936	0.940	0.931	0.929	0.915
Perindopril MPO	score	0.778	0.811	0.773	0.790	0.779	0.764	0.758	0.780	0.779	0.774
	similarity	0.629	0.593	0.627	0.573	0.589	0.628	0.633	0.628	0.627	0.594
	top1	0.779	0.816	0.775	0.791	0.784	0.767	0.758	0.780	0.784	0.777

	score	0.919	0.915	0.919	0.879	0.913	0.915	0.918	0.913	0.919	0.918
Amlodipine MPO	similarity	0.679	0.684	0.695	0.677	0.677	0.684	0.685	0.675	0.695	0.689
	top1	0.924	0.923	0.924	0.906	0.923	0.923	0.924	0.923	0.924	0.924
	score	0.911	0.939	0.942	0.910	0.925	0.928	0.911	0.944	0.930	0.936
Sitagliptin MPO	similarity	0.176	0.240	0.281	0.275	0.304	0.246	0.212	0.266	0.261	0.282
	top1	0.927	0.948	0.945	0.931	0.934	0.945	0.926	0.948	0.942	0.939
	score	0.659	0.633	0.643	0.628	0.637	0.640	0.628	0.631	0.643	0.638
Zaleplon MPO	similarity	0.341	0.377	0.418	0.363	0.316	0.350	0.298	0.363	0.396	0.367
	top1	0.695	0.668	0.656	0.647	0.668	0.668	0.668	0.653	0.668	0.668
	score	0.960	0.979	0.961	0.961	0.983	0.957	0.972	0.932	0.952	0.925
Valsartan SMARTS	similarity	0.261	0.289	0.295	0.244	0.276	0.229	0.242	0.238	0.232	0.240
	top1	0.999	0.999	0.989	0.994	0.996	0.987	0.993	0.982	0.991	0.985
	score	1.0	1.0	1.0	1.0	1.0	1.0	1.0	1.0	1.0	1.0
Deco Hop	similarity	0.384	0.417	0.387	0.427	0.469	0.403	0.357	0.376	0.420	0.434
	top1	1.0	1.0	1.0	1.0	1.0	1.0	1.0	1.0	1.0	1.0
	score	0.928	0.919	0.926	0.930	0.925	0.916	0.924	0.927	0.939	0.935
Scaffold Hop	similarity	0.366	0.310	0.387	0.365	0.295	0.288	0.353	0.401	0.346	0.295
	top1	0.943	0.934	0.936	0.948	0.937	0.933	0.936	0.939	0.950	0.945
